# Time-Dependent Progression of Demyelination and Axonal Pathology in MP4-Induced Experimental Autoimmune Encephalomyelitis

**DOI:** 10.1371/journal.pone.0144847

**Published:** 2015-12-11

**Authors:** Johanna Prinz, Aylin Karacivi, Eva R. Stormanns, Mascha S. Recks, Stefanie Kuerten

**Affiliations:** 1 Department of Anatomy I, University of Cologne, Joseph-Stelzmann-Str. 9, 50931, Cologne, Germany; 2 Department of Anatomy II, University of Cologne, Joseph-Stelzmann-Str. 9, 50931, Cologne, Germany; 3 Department of Anatomy and Cell Biology, University of Würzburg, Koellikerstraße 6, 97070, Würzburg, Germany; Julius-Maximilians-Universität Würzburg, GERMANY

## Abstract

**Background:**

Multiple sclerosis (MS) is an autoimmune disease of the central nervous system (CNS) characterized by inflammation, demyelination and axonal pathology. Myelin basic protein/proteolipid protein (MBP-PLP) fusion protein MP4 is capable of inducing chronic experimental autoimmune encephalomyelitis (EAE) in susceptible mouse strains mirroring diverse histopathological and immunological hallmarks of MS. Limited availability of human tissue underscores the importance of animal models to study the pathology of MS.

**Methods:**

Twenty-two female C57BL/6 (B6) mice were immunized with MP4 and the clinical development of experimental autoimmune encephalomyelitis (EAE) was observed. Methylene blue-stained semi-thin and ultra-thin sections of the lumbar spinal cord were assessed at the peak of acute EAE, three months (chronic EAE) and six months after onset of EAE (long-term EAE). The extent of lesional area and inflammation were analyzed in semi-thin sections on a light microscopic level. The magnitude of demyelination and axonal damage were determined using electron microscopy. Emphasis was put on the ventrolateral tract (VLT) of the spinal cord.

**Results:**

B6 mice demonstrated increasing demyelination and severe axonal pathology in the course of MP4-induced EAE. In addition, mitochondrial swelling and a decrease in the nearest neighbor neurofilament distance (NNND) as early signs of axonal damage were evident with the onset of EAE. In semi-thin sections we observed the maximum of lesional area in the chronic state of EAE while inflammation was found to a similar extent in acute and chronic EAE. In contrast to the well-established myelin oligodendrocyte glycoprotein (MOG) model, disease stages of MP4-induced EAE could not be distinguished by assessing the extent of parenchymal edema or the grade of inflammation.

**Conclusions:**

Our results complement our previous ultrastructural studies of B6 EAE models and suggest that B6 mice immunized with different antigens constitute useful instruments to study the diverse histopathological aspects of MS.

## Introduction

Multiple sclerosis (MS) is thought to be a chronic autoimmune disease of the central nervous system (CNS). For decades research has focused on identifying the mechanisms underlying demyelination and multifocal centers of inflammation in the white matter of the CNS [1–4]. Due to the limited availability of human CNS tissue animal models for MS are frequently used to perform mechanism-oriented studies [2, 5, 6]. One of the common models for MS is experimental autoimmune encephalomyelitis (EAE), in which susceptible animal strains are immunized with CNS antigens [7–10]. Originally, spinal cord homogenate (SCH) was used to induce EAE [11]. Today single proteins or peptides derived from those proteins in particular from the myelin sheath are typically employed. Among these proteins are myelin basic protein (MBP), proteolipid protein (PLP) or myelin oligodendrocyte glycoprotein (MOG) [1, 12–16]. None of the available EAE models can entirely mirror the immunopathology of MS on its own, but each model displays differential aspects of the disease entity. We have recently introduced a new model on the C57BL/6 (B6) background, the MBP-PLP fusion protein (MP4)-induced EAE, and we have distinguished several advantages of working with this antigen [16]. Among these advantages were the chronic disease course and the dependence on B cells and antibodies [17, 18]. Our studies have also shown fundamental differences in CNS histopathology when comparing MP4-induced EAE and the traditional MOG peptide 35–55 model with regard to the topography of lesions and the composition of CNS infiltrates [17, 19].

We have recently also initiated ultrastructural studies of MP4-induced EAE that were focused on the dorsal corticospinal tract (CST) and motor neuron damage. We have demonstrated that MP4-induced EAE was characterized by CST degeneration and alterations in the motor neuron perikaryal phosphorylation status [20]. There was an association between the clinical disease severity and the extent of CST degeneration [20]. These data were in parallel with results obtained in MS that demonstrated a relationship between the progression of neurodegeneration and disability [21, 22].

Our current study pays particular attention to the pathological changes in the ventrolateral tract (VLT) of the lumbar spinal cord. We investigated the morphological correlates of inflammation, demyelination and axonal damage studying both methylene blue-stained semi-thin sections and electron microscopic images. Our data show differential patterns of inflammation, demyelination and axonal damage in the course of MP4-induced EAE. In particular in comparison to both the chronic MOG:35–55 and the relapsing-remitting PLP:139–151 model that we have already characterized in detail, this study should provide valuable insights into the impact of immunization with different CNS antigens on the resulting CNS pathology.

## Materials and Methods

### Mice

Twenty-two six- to eight-week-old female wild-type (WT) B6 mice were obtained from Janvier Labs (Saint Berthevin Cedex, France) and kept under specific-pathogen conditions in the animal facilities of the Department of Anatomy of the University of Cologne, Germany. Mice were maintained in individually ventilated cages with autoclaved woodchip bedding in groups of two to five mice. Mice were fed a standard rodent diet (Altromin Spezialfutter GmbH & Co. KG, Lage, Germany) and had free access to pathogen-free water. From the time when mice displayed paralytic signs feed and water were offered at ground level. All animal experiments complied with the German Law on the Protection of Animals and the ‘‘Principles of laboratory animal care” (NIH publication No. 86–23, revised 1985) and the ARRIVE guidelines (S1 Appendix). The treatments were performed according to a protocol that was approved by the LANUV, Germany (approval number 2011.A276).

### Induction and assessment of EAE

MP4 was obtained from Alexion Pharmaceuticals (Cheshire, CT). Incomplete Freund's adjuvant (IFA) was prepared as a mixture of mannide monooleate (Sigma-Aldrich, St. Louis, MO) and paraffin oil (EM Science, Gibbstown, NJ), and complete Freund's adjuvant (CFA) was obtained by mixing *Mycobacterium tuberculosis* H37 Ra (Difco Laboratories, Franklin Lakes, NJ) at 5 mg/ml into IFA. For disease induction, mice were immunized subcutaneously in both sides of the flank with 200 μg MP4 (stock: 2 mg/ml) in CFA. Control mice remained untreated. On the day of immunization and 48 h later, 200 ng pertussis toxin (PTX; List Biological Laboratories, Hornby, ONT, Canada) were given in 500 μl sterile phosphate-buffered saline (PBS). The development of EAE was evaluated daily and the paralytic signs were classified following the standard scale: (0), no disease; (1), floppy tail; (2), hind limb weakness; (3), full hind limb paralysis; (4), quadriplegia; (5), death. Mice that were in between the clear-cut gradations of clinical signs were scored intermediate in increments of 0.5. Any mouse which was scored as a grade of 4 for more than 48 h was euthanized. Table 1 shows the classification of mice used in this study according to disease stage and EAE score.

**Table 1:** Number of mice in each group and mean value of the clinical score ± SD.

Disease stage	Time point of testing	Number of mice	EAE score ± SD
**Acute**	peak of acute EAE	7	1.78 ± 0.75
**Chronic**	three months after onset of EAE	5	1.7 ± 0.57
**Long-term**	six months after onset of EAE	10	1.72 ± 0.53

### Analysis of methylene blue-stained spinal cord sections

For assessment of spinal cord histopathology, three transverse segments from the lumbar region were obtained from each mouse at the peak of acute paralytic signs (acute EAE), three months after onset of EAE (chronic EAE) and six months after onset of EAE (long-term EAE). Mice were sacrificed with CO_2_ and perfused intracardially with 4% paraformaldehyde and 4% glutaraldehyde in 0.1M PBS (pH 7.4). Specimens were post-fixed at 4°C for at least 24 h before spinal cords were carefully removed from the vertebral canal. Samples were rinsed in 0.1M cacodylate buffer (pH 7.35) three times and fixed in 1% osmium on ice for 4 h. The tissue was then treated with 1.5% uranyl acetate in 70% ethanol for contrast enhancement and dehydrated in a graded series of ethanol at −20 C°. Sections were embedded in epon (Fluka, St. Louis, MO) and polymerized at 60°C for at least three days. One μm thick semi-thin transverse sections were then cut on a Leica Ultracut UCT ultramicrotome (Leica Microsystems, Wetzlar, Germany) and stained with methylene blue. Ten to 25 images from 22 mice with two to four sections per mouse were evaluated using a light microscope (Leica DM LB2, Leica Microsystems, Wetzlar, Germany). Images were acquired using Zeiss AxioCam MRc and AxioVision 40 4.7 software (Carl Zeiss AG, Oberkochen, Germany). Subsequently, Image Pro Plus 6.0 software (Version 6.0, Media Cybernetics, Bethesda, MD, USA) was used to measure the size of the ventrolateral, the posterior and the corticospinal tract. Additionally, the percentage of the lesional area in each tract of the spinal cord was measured. The extent of parenchymal inflammation was assessed according to a semi-quantitative scale ranging from 0 (no cellular infiltration) to 5 (extensive cellular infiltration). The grade of edema was classified simultaneously using a semi-quantitative scale ranging from 0 (no edema) to 6 (extensive edema).

### Electron microscopic assessment

The evaluation of 80 nm thick ultra-thin sections was performed on a Zeiss EM 902 transmission electron microscope (Carl Zeiss AG, Oberkochen, Germany) and images were captured from the center of the lesional area in the VLT with a digital EM camera (MegaView III, Olympus Soft Imaging Systems GmbH, Münster, Germany). For the examination of acute EAE 21 sections of seven mice were investigated. In addition, 16 sections of five mice in the chronic stage and 23 sections of 10 mice with long-term EAE were analyzed. Seven images at 7000× magnification were acquired from each spinal cord section and the total number of nerve fibers/mm^2^ was counted. Histological examination of myelin and axonal pathology was done blinded as to the clinical score and disease stage of the animals.

#### Myelin pathology

Image-Pro Plus software was used to count the number of normal appearing, completely demyelinated, or remyelinated nerve fibers, respectively, in addition to those in the process of demyelination. The *g-ratio* was used for the distinction between the different categories [23, 24], and calculated by dividing the diameter of the axon by the diameter of the whole nerve fiber. The optimal *g-ratio* for the ventrolateral column was set between 0.72 and 0.81 [23]. Axons showing a *g-ratio* below this level were characterized as *demyelinating*. Axons showing a *g-ratio* above this level were characterized as *remyelinating*. The term *physiological* was applied to axons with an intact myelin sheath and no other signs of axonal damage.

#### Axonal damage

Analysis of axonal damage comprised the assessment of complete axolysis, a decrease of the nearest neighbor neurofilament distance (NNND) [25] and mitochondrial swelling [26, 27]. The size of all mitochondria within an axon was set in relation to the total area of each axoplasm (*mito-ratio*). The cut-off value for mitochondrial swelling was set at 0.149 following our previously established analyses [28]. To achieve an objective evaluation of the NNND, the distance between the single neurofilaments was measured and the cut-off value for a decrease in NNND was set at 0.039 μm [28]. While complete degeneration of axons (axolysis) was defined as *gross* axonal pathology, mitochondrial swelling and a NNND decrease were designated as *fine* axonal damage [25, 27, 29]. The extent of myelin and axonal pathology as described above was assessed for each nerve fiber and the number of fibers with isolated myelin or axonal damage or combined pathology was evaluated.

### Statistical analysis

SigmaPlot software (Version 12.0, Systat Software, San Jose, CA, USA) was used for statistical analysis. In case of a normal distribution, differences between groups were assessed using Student`s t-test. The Mann-Whitney U rank-sum test was applied in case the normality test failed. Three levels of statistical significance were differentiated: *p ≤ 0.05, **p < 0.01 and ***p < 0.001. Spearman`s correlation was used to assess the correlation between axolysis and mitochondrial damage or axolysis and a decreased NNND, respectively.

## Results

### Inflammation and neurodegeneration coexist in the course of MP4-induced EAE

**Fig 1:**
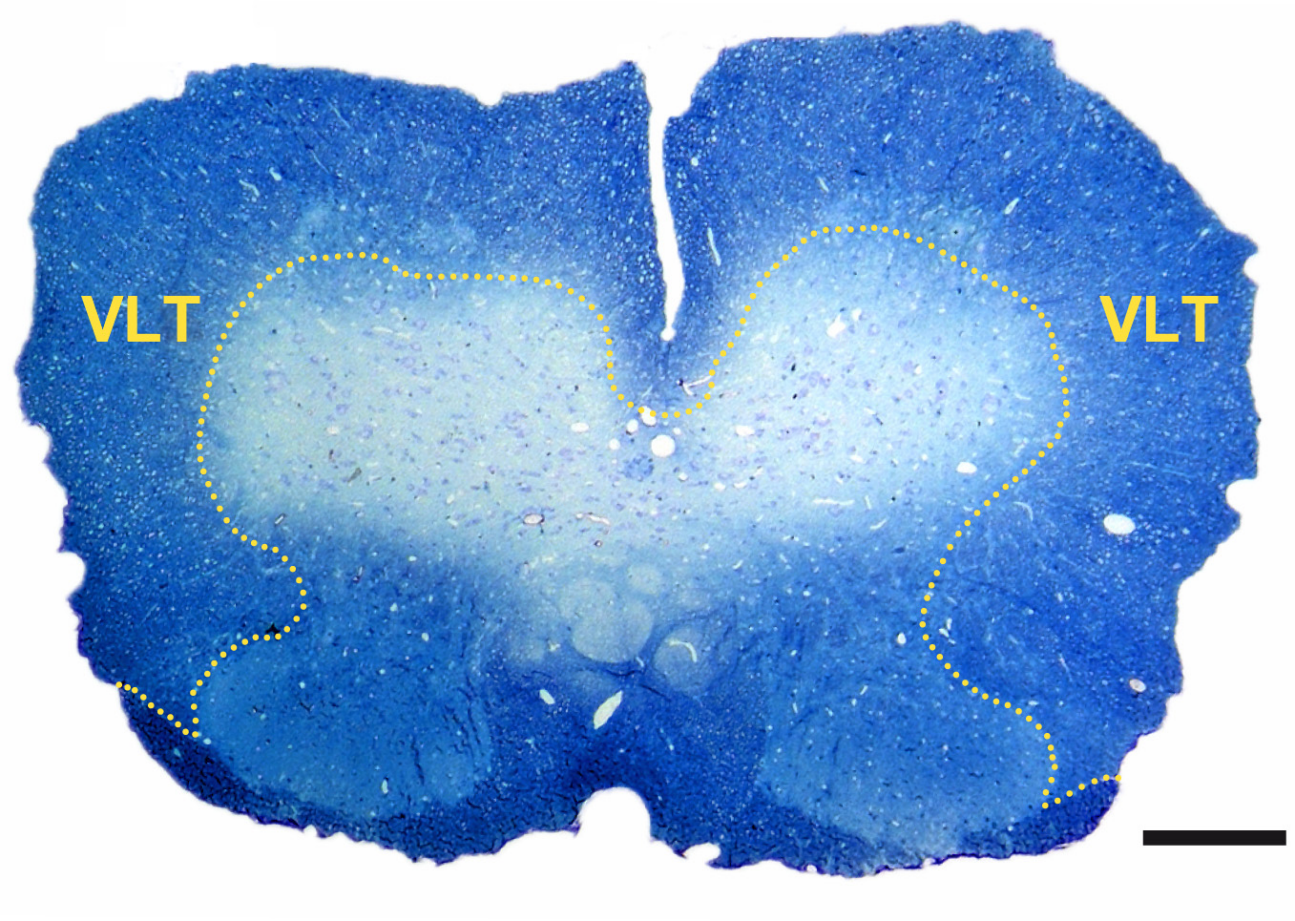
Localization of the VLT in the murine spinal cord. The image shows a methylene blue-stained transverse section of murine lumbar spinal cord in a MP4-immunized mouse in the long-term stage of the disease. The VLT is circled. The scale bar depicts 200 μm.

**Fig 2:**
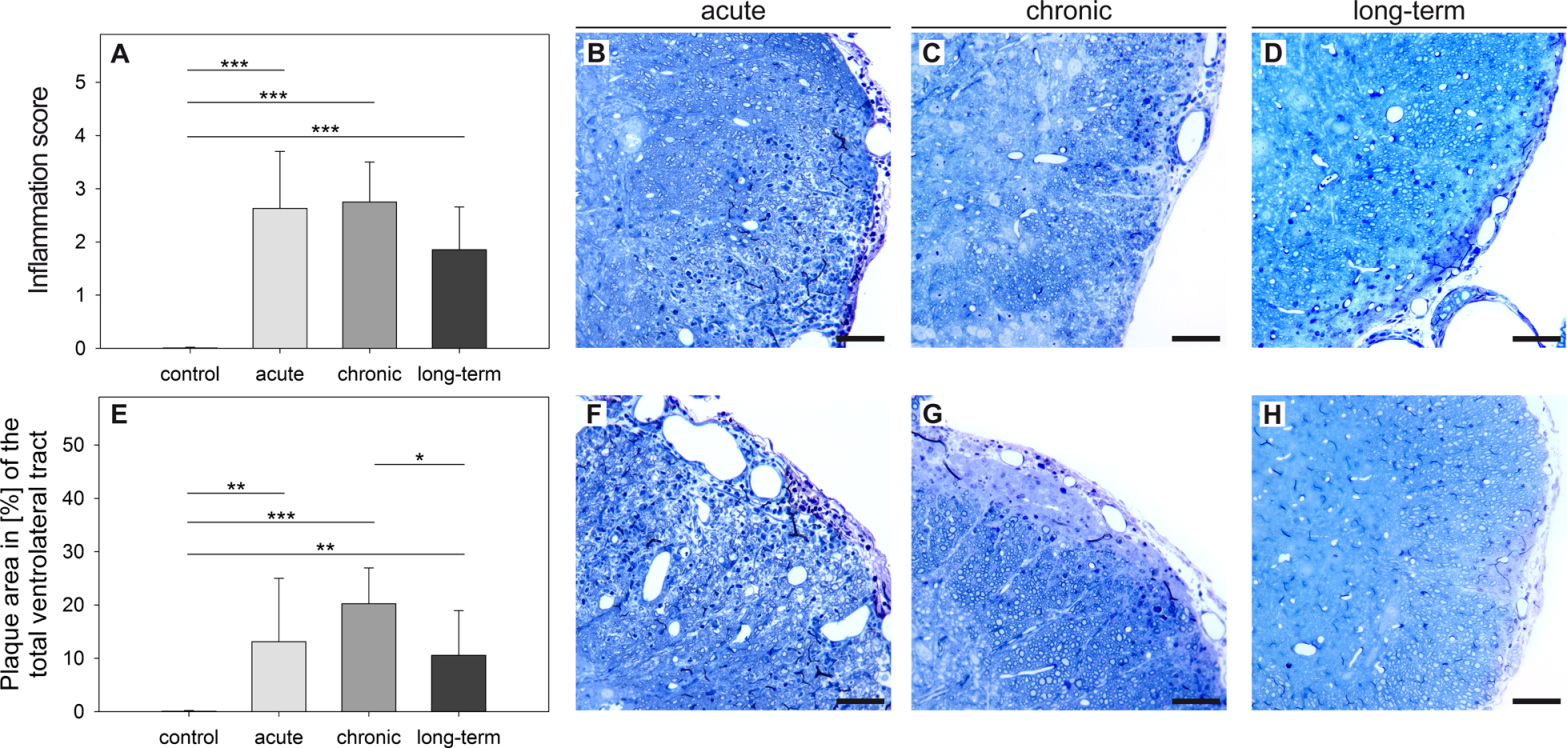
Time-dependent alterations of inflammation and lesional area in MP4-induced EAE. Semi-thin sections of the VLT were obtained from B6 mice in acute EAE, three months (chronic) and six months after EAE onset (long-term). Sections were stained with methylene blue and compared to a non-immunized control group that consisted of *n =* 10 mice. Cellular infiltration (A) was graded on a semi-quantitative scale and the lesional area size was measured (E). The means ± SD are shown for a total number of *n =* 7 mice in the acute, *n =* 5 mice in the chronic and *n =* 10 mice in the long-term stage of EAE. Representative images from every stage of the disease are displayed showing cellular inflammation (B-D) and the lesional area (F-H). *p ≤ 0.05; **p ≤ 0.01; ***p ≤ 0.001. Scale bars depict 50 μm.

Fig 1 delineates the localization of the VLT in a lumbar section of the murine spinal cord. A semi-quantitative scoring system as described in *Materials and methods* was used to analyze the dimension of inflammation and edema in MP4-induced EAE. The size of the lesional area was determined as a morphological correlate of neurodegeneration. The mean pathological score of inflammation and the mean value of the spinal cord lesional area expressed as percentage are shown in Fig 2A and 2E and representative images are demonstrated in Fig 2B–2D and 2F–2H. Inflammation was found to a similar extent in the acute and chronic stage of EAE, while it decreased in the long-term stage of the disease. Of note, in contrast to the MOG:35–55 model [28, 30] the spinal cord of B6 mice immunized with MP4 hardly showed any parenchymal edema, while its extent was still slightly above the level of the control mice (data not shown). The lesional area significantly increased from acute to chronic EAE and again decreased in the long-term stage of EAE.

### Progressive myelin pathology is associated with the disease kinetics in MP4-induced EAE

**Fig 3:**
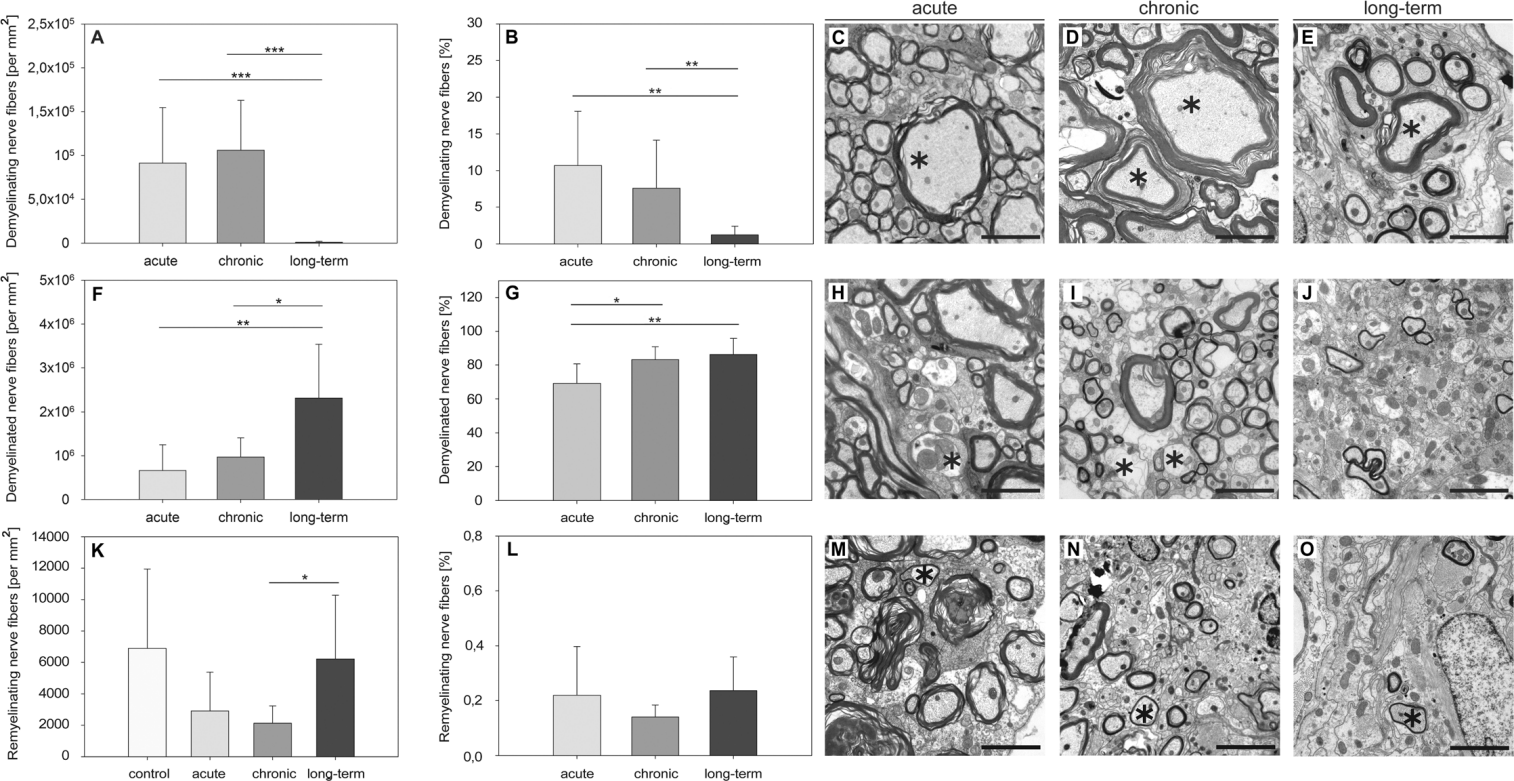
Differences in myelin pathology in the course of MP4-induced EAE. Ultra-thin spinal cord sections were obtained from MP4-immunized B6 mice in different disease stages and images were taken from the center of the lesional area in the VLT. The numbers and percentages of nerve fibers being either in the process of demyelination (A, B), completely demyelinated (F, G) or remyelinated (K, L) were evaluated. Values obtained from non-immunized control mice were subtracted for the nerve fibers being in the process of demyelination and for completely demyelinated nerve fibers. The means ± SD are demonstrated for a total number of *n =* 7 control mice, *n =* 7 mice in the acute, *n =* 5 mice in the chronic and *n =* 10 mice in the long-term stage of the disease. Representative images from every stage of EAE depicting demyelinating (C-E), demyelinated (H-J) or remyelinating nerve fibers (M-O) are displayed. *p ≤ 0.05; **p ≤ 0.01; ***p ≤ 0.001. Scale bars depict 10 μm.

In the context of an autoimmune disease such as MS, several self-antigens are affected. In MS and EAE these targets typically belong to the myelin sheath and include MBP, MOG, PLP, myelin-associated glycoprotein (MAG) or neuronal structures [31]. Nerve fibers being in the process of demyelination morphologically differ from physiological axons. The myelin sheath shows swelling and myelin lamellae begin to diverge widely from each other [32, 33]. Representative images are shown in Fig 3. We evaluated ultrastructural images of the VLT at 7000× magnification and classified each axon as being in the process of demyelination or completely demyelinated. For this purpose we assessed the *g-ratio* as described in *Materials and methods*. The following aspects were remarkable. On the one hand, we found a high number of axons in the process of demyelination in the acute stage and this number further increased slightly in chronic EAE (Fig 3A–3E). In long-term EAE significantly less demyelinating fibers were observed (Fig 3A–3E). In contrast, complete degeneration of the myelin sheath was evident throughout the course of the disease and reached a maximum in the long-term stage (Fig 3F–3J). Axons displaying isolated myelin damage (i.e. without any signs of axonal pathology) were already evident in the acute stage of EAE and their numbers significantly increased with the progression of the disease (acute 568,691.42 ± 421,727.94 per mm^2^, chronic 888,207 ± 318,821.72 per mm^2^ and long-term 1,495,870.6 ± 764,138.56 per mm^2^, with acute *vs* chronic: *p* = 0.164, chronic *vs* long-term: *p* = 0.067 and acute *vs* long-term: *p* = 0.027).

### There was no evidence of spinal cord remyelination associated with MP4-induced EAE

Remyelinated nerve fibers are characterized by dense and thin myelin lamellae [34]. We evaluated ultrastructural images of the VLT at 7000× magnification and detected nerve fibers that were in the state of regeneration (Fig 3K and 3L). The data show that both demyelination and remyelination coexisted at the same time. The occurrence of remyelinated nerve fibers was not just restricted to the long-term stage of EAE, but was also present in the acute and chronic state of the disease. However, the extent of remyelinating nerve fibers was below the level that was evident in non-immunized control mice. Expressed as a percentage we only found 0.21% ± 0.17% remyelinating nerve fibers in the acute, 0.14% ± 0.04% in the chronic and 0.23% ± 0.12% in the long-term stage of EAE. Fig 3M–3O shows representative images of remyelinating nerve fibers in every stage of the disease.

### The maximum of axonal damage was observed in long-term EAE

**Fig 4:**
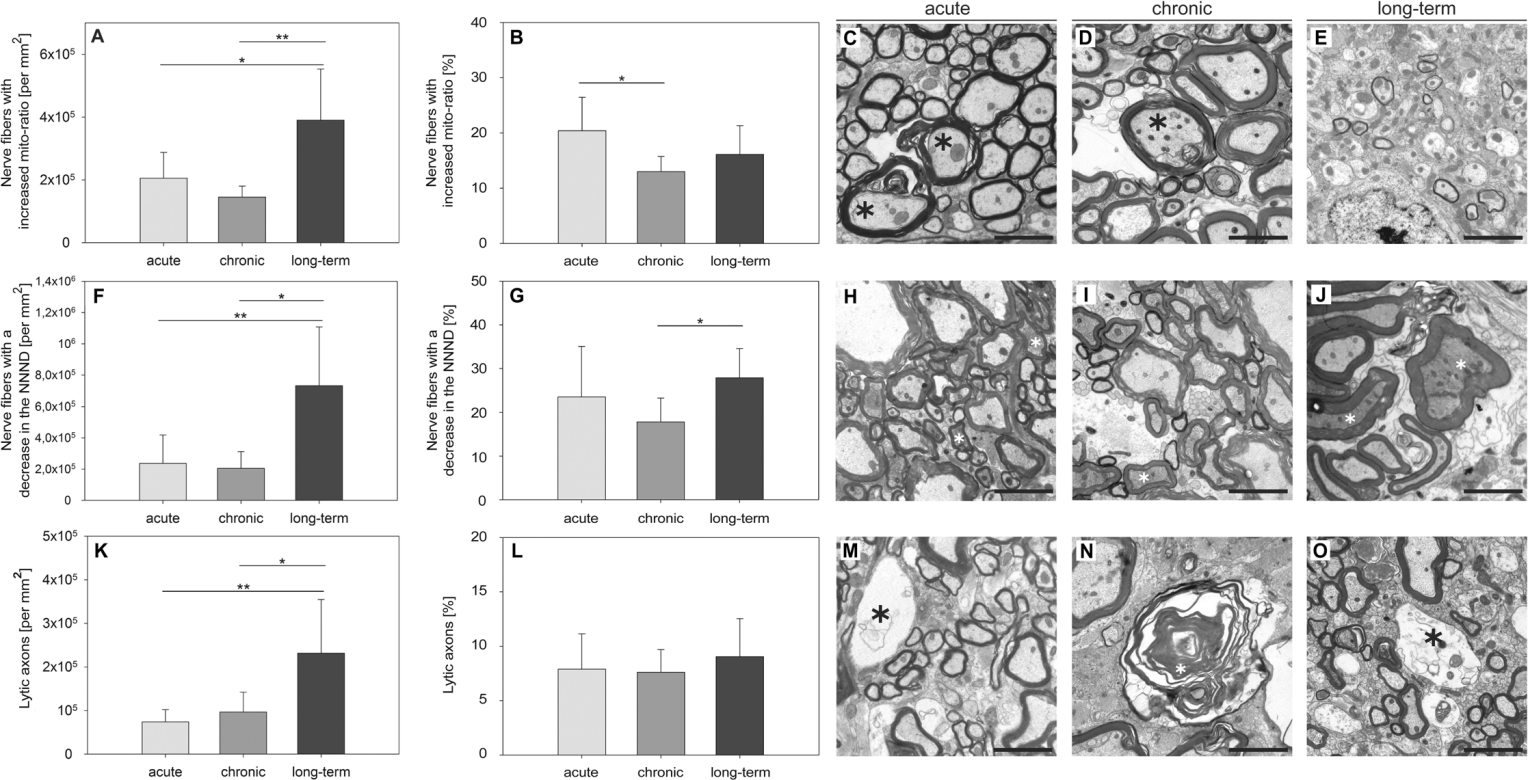
The extent of axonal pathology peaks six months after the onset of EAE in MP4-immunized B6 mice. Ultra-thin spinal cord sections were obtained and images were taken from the center of the lesional area in the VLT. The numbers and percentages of axons showing mitochondrial swelling (A, B), a decreased NNND (F, G) or complete axolysis (K, L) were evaluated. Values obtained from non-immunized control mice were subtracted from the absolute number. The means ± SD are demonstrated for a total of *n =* 7 mice with acute, *n =* 5 mice with chronic and *n =* 10 mice with long-term EAE. Representative images obtained in each disease stage are providing examples of mitochondrial swelling (C-E), a decrease in the NNND (H-J) and axolysis (M-O). *p ≤ 0.05; **p ≤ 0.01; ***p ≤ 0.001. Scale bars depict 10 μm.

**Fig 5:**
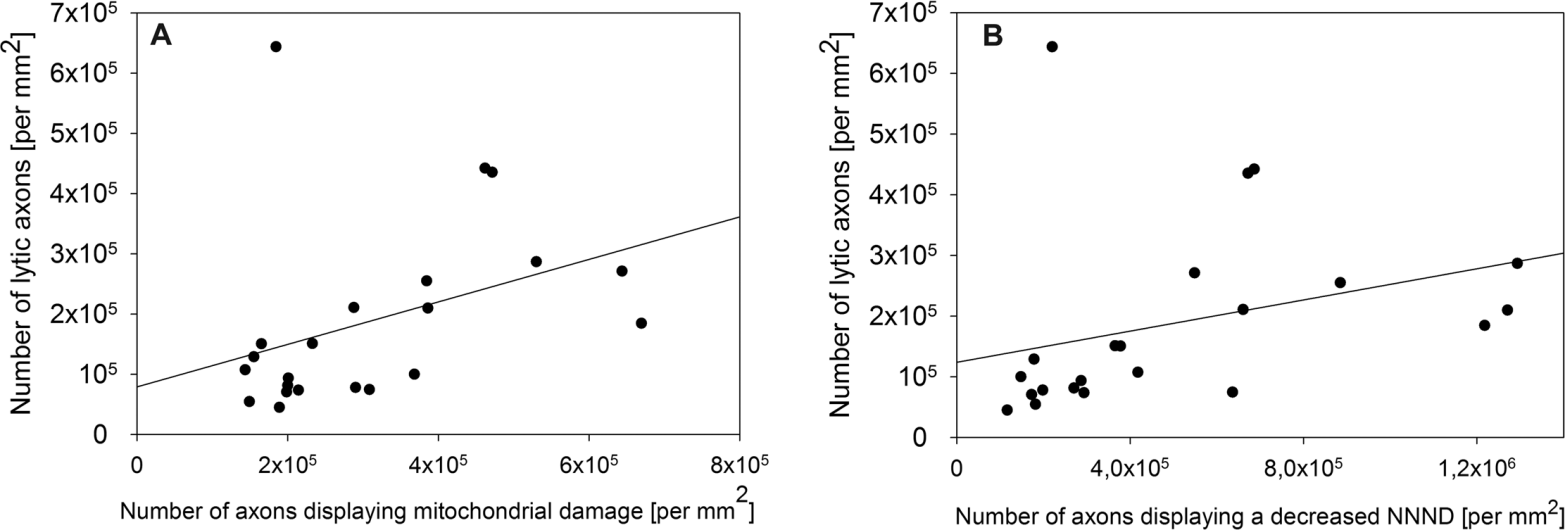
Correlation between axolysis and mitochondrial swelling or axolysis and a decreased NNND, respectively. (A) Significant correlation between the number of lytic axons and the number of axons showing mitochondrial swelling (*r*_*s*_ = 0.519 and *p* = 0.0135). (B) Significant correlation between the number of lytic axons and the number of axons with a decreased NNND (*r*_*s*_ = 0.660 and *p* = 0.000792). Results refer to *n* = 22 MP4-immunized mice.

We examined the axoplasm at 7000× magnification putting emphasis on axolysis, mitochondrial swelling and a decrease in the NNND as morphological correlates for axonal damage [25, 26]. Mitochondrial swelling in relation to the total axonal area was measured and the *mito-ratio* was determined. A significant number of axons with an increased *mito-ratio* was observed in each stage of EAE (Fig 4A–4E). Next, we analyzed the NNND as described in *Materials and methods* and measured the distance between the single neurofilaments in μm whose decrease is considered to be an early sign of neurodegeneration [25]. A significant number of axons with a decreased NNND was evident in the course of EAE (Fig 4F–4J). In addition, we examined the amount of axolysis as a correlate of irreversible axonal damage. Fig 4K–4O show that axolysis already existed in an early stage of MP4-induced EAE and significantly increased with disease progression. There was a significant correlation between axolysis and a decrease in the NNND (*r*_*s*_ = 0.660 and *p* = 0.0008) or axolysis and mitochondrial swelling, respectively (*r*_*s*_ = 0.519 and *p* = 0.0135, Fig 5A and 5B). Isolated axonal damage (i.e. without evidence of myelin pathology) was already observed in the early stage of MP4-induced EAE and significantly increased in long-term disease (acute 71,747.42 ± 34,588.28 per mm^2^, chronic 27,529.8 ± 11,802.62 per mm^2^ and long-term 121,344.9 ± 33,316.92 per mm^2^, with acute *vs* long-term: *p* = 0.009 and chronic *vs* long-term: *p* < 0.001). The combination of both myelin and axonal pathology first reached a plateau within the first three months of EAE before it further significantly increased six months after the onset of the disease (acute 465,760.42 ± 206,287.55 per mm^2^, chronic 468,415 ± 142,799.45 per mm^2^ and long-term 1,082,142.7 ± 530,122.17 per mm^2^, with acute *vs* long-term: *p* = 0.011 and chronic *vs* long-term: *p* = 0.027). In relation to the total number of axons we found 47.5% ± 12.4% isolated myelin damage in the acute, 63.2% ± 7.2% in the chronic and 53.6% ± 8.3% in the long-term stage of EAE. Isolated axonal damage was observed to a lower extent in the acute 8.0% ± 5.5% and chronic stage 2.3% ± 1.7% and six months after the onset of EAE 6.4% ± 5.1%. In the acute stage, 44.5% ± 12.5% nerve fibers showed the combination of both myelin and axonal damage compared to 34.5% ± 6.6% in the chronic and 40% ± 6.6% in the long-term stage of the disease.

## Discussion

MS is characterized by inflammation, demyelination and axonal damage [3, 4, 35]. In particular autoreactive T cells, activated microglia and macrophages are thought to constitute a major part of the inflammatory component [36, 37]. Only recently, however, B cell aggregates were found in the meninges of patients with secondary progressive MS and associated with more severe cortical pathology [38, 39]. The limited availability of human CNS tissue makes it necessary to focus research on suitable animal models in order to get further insights into the pathology underlying irreversible functional deficits in patients. One of the most commonly used animal models is EAE, which is elicited by immunization of susceptible mouse strains with CNS antigens or by the passive transfer of encephalitogenic T cells [2]. There are also spontaneous models that rely on genetic manipulations of the T cell receptor [40]. The genetic background of the animal strain and the antigens used for disease induction have an impact on the clinical course of the disease and the histopathological features of each model [41].

In our previous studies we compared the clinical outcome, cellular composition of CNS infiltrates and features of neurodegeneration in B6 mice immunized with PLP:178–191, MOG:35–55 or MP4 [16, 17, 42]. PLP:178-191-elicited EAE was characterized by a delayed EAE onset and showed a chronic disease course [19]. Further studies of this model showed a loss of axonal density in the VLT in the absence of pathological alterations of the dorsal tract or the motor neurons. Additionally, we did not observe substantial demyelination in the course of the disease [42]. B6 mice immunized with other PLP peptides such as PLP:43–64, PLP:50–64 or PLP:94–108 did not display demyelination either [43]. Therefore and because of the difficulty of working with the whole PLP protein, studies that rely on PLP-induced EAE rather play a minor role in our current understanding of MS pathogenesis. In contrast, MOG:35–55 is one of the most frequently used EAE antigens that causes chronic disease, extensive spinal cord inflammation and demyelination in B6 mice [13, 44]. While inflammation could be found only in early stages of MOG:35-55-induced EAE, myelin damage was consistent throughout the disease, but significantly decreased with disease progression [28]. No significant differences in the composition of cellular infiltration could be found comparing the acute and chronic stage of MOG:35-55-induced EAE [17]. Our previous thorough histological characterization of PLP:178-191- and MOG:35-55-induced EAE on the B6 background enabled us to make direct comparisons with MP4-immunized EAE in the current study.

Our previous research has provided clear evidence that MP4-induced EAE is B cell-dependent, not only because B cell-deficient mice were resistant to EAE induction [16], but also a remarkable amount of B cells was found within the CNS infiltrates, which formed B cell aggregates and tertiary lymphoid organs as disease progressed [45]. Evidence that B cells are crucially involved in the pathogenesis of MS is accumulating and has lead to the implementation of new B cell-specific therapies. The monoclonal chimeric antibody rituximab (Mabthera®) is directed against the B cell CD20 antigen and was originally approved for the treatment of non-Hodgkin`s lymphoma (NHL) in 1997 [46]. In patients with MS rituximab induces a long-lasting depletion of the CD20^+^ B cells. Hauser et al. demonstrated that patients with relapsing-remitting MS (RRMS) treated with rituximab showed a reduced number of gadolinium-enhancing lesions as detected by magnetic resonance imaging (MRI) scans of the brain [47]. The monoclonal anti-CD52 antibody alemtuzumab (MabCampath®) depletes CD52 expressing B cells, T cells, monocytes and macrophages without affecting stem cells [48]. Comparing interferon-beta 1a treatment of RRMS to alemtuzumab over two years showed that only 22% of patients treated with alemtuzumab developed a relapse compared to nearly 60% of the patients treated with interferon-beta 1a. [49]. Another novel monoclonal antibody for the treatment of MS is the humanized anti-CD20 antibody ocrelizumab [50]. Patients receiving ocrelizumab in a phase II trial had up to 96% lower gadolinium-enhancing MRI lesions and relapse rates were up to 80% lower than in the placebo and an interferon-beta 1a group [51]. Ocrelizumab will probably be approved for the treatment of MS in the beginning of 2016.

In contrast to the MOG:35–55 model MP4 triggered an initial wave of immune cell infiltration into the brain, which disappeared in the course of EAE and shifted to the cerebellar white matter [19]. Therefore, not only the comparison of different EAE models, but also of different CNS regions allowed conclusions as to distinct patterns of inflammation, demyelination and nerve fiber damage [20]. Studying the CST of MP4-immunized B6 mice we found not only axonal damage and myelin degeneration, but also identified differences in the degree of motor neuron perikaryal phosphorylation as a morphological correlate for gray matter damage [20]. In the same model we observed severe nuclear membrane defects and a decrease in the quantity of synapses in addition to a beginning disintegration of the rough endoplasmatic reticulum [20]. In our present study we focused our analysis on the VLT accounting for the largest part of the murine spinal cord. So far, previous studies on MP4-induced EAE have not dealt with an ultrastructural characterization and it was our aim to delineate whether this model was a valuable tool for ultrastructural studies of MS histopathology. The assessment of semi-thin sections demonstrated that similar to the MOG:35–55 model inflammation was present within the first three months after the onset of the disease. However, in contrast to the MOG:35–55 model, which showed a continuous decrease of inflammation with disease progression [30], we observed that inflammation was nearly in a steady state in the course of MP4-induced EAE. Another remarkable difference between the two animal models was the extent of the parenchymal edema. Analyzing the VLT of the MP4 model, hardly any edema could be detected, while in the MOG:35–55 model marked edema was observed at least in the beginning of the EAE [30]. The analysis of regeneration demonstrated a significant increase in the number of remyelinated nerve fibers in the MOG:35–55 model, in particular in the long-term stage of EAE. Furthermore, remyelination was found to a greater extent in the VLT than in the dorsolateral tract of the spinal cord and was significantly increased compared to non-immunized control mice [28]. In MP4-induced EAE the numbers of remyelinating nerve fibers were below the numbers measured in non-immunized control mice, which suggests that this model is characterized by irreversible spinal cord damage. Comparing the MOG:35–55 and the MP4 model in regard to spinal cord regeneration might be a suitable tool to study mechanisms of neurodegeneration and–regeneration and treatment options addressing this aspect of MS pathology.

In MP4-induced EAE both demyelination and axolysis could be detected with the onset of the disease. These data are in line with our previous ultrastructural studies of the MOG:35–55 model that showed axonal pathology in the VLT from the earliest disease stages on [28]. Similarly, analyses of biopsy and autopsy tissues from the CNS of patients demonstrated demyelination and axonal transection in early disease stages [4, 21]. Our results also show that the numbers of completely demyelinated axons increased in the course of the disease. Simultaneously with this increase in demyelinated nerve fibers the numbers of lytic axons increased significantly underlining the traditional notion that axonal injury is the result of demyelination [4]. Furthermore, we observed an elevated number of axons with a decreased NNND. A decrease in the NNND, which depends on the phosphorylation status of neurofilament sidearms, is considered to coincide with a loss of myelination [21, 25, 52, 53]. Research on *post mortem* human CNS tissue showed an increase of dephosphorylated neurofilaments in MS patients while a high amount of hyperphosphorylated neurofilaments could be detected in acute lesions [53], representing the changing dynamics of axonal pathology in the course of MS. Ultrastructural examinations of the CST and motor neurons in B6 mice immunized with MOG:35–55 or MP4, respectively, demonstrated a decrease in the NNND in both models. Another feature of axonal pathology that we investigated in the present study was the extent of mitochondrial swelling. Here we show that there was no significant degree of mitochondrial swelling in the beginning of EAE, while it was evident with the transition to chronic disease. In contrast to this finding mitochondrial swelling could be detected early on in the MOG:35–55 model [28]. Both features, a decrease in the NNND and mitochondrial swelling are known to be early signs of axonal damage [25–27]. Accordingly, we found a significant correlation between axolysis and both a decreased NNND and mitochondrial swelling.

Another well-established animal model is the non-obese diabetic (NOD) mouse that develops spontaneous diabetes. This mouse strain was formerly developed to establish a cataract-prone subline (CTS) and it is the most common model for investigating the human autoimmune type I diabetes [54–56]. Immunization of NOD mice with MOG:35–55 results in an initial acute EAE attack, followed by a progressively worsening relapse mirroring secondary progressive MS (SPMS) [57]. Mayo et al. have created a hybrid mouse strain by cross-breeding NOD and B6 mice. These mice showed chronic progressive EAE after immunization with MOG:35–55 [58]. The introduction of a MP4/NOD model may be an additional valuable tool to further investigate the histopathological hallmarks of progressive MS.

## Supporting Information

S1 Appendix ARRIVE guidelines. (PDF)
