# Correction: Time-Dependent Progression of Demyelination and Axonal Pathology in MP4-Induced Experimental Autoimmune Encephalomyelitis

**DOI:** 10.1371/journal.pone.0155197

**Published:** 2016-05-04

**Authors:** Johanna Prinz, Aylin Karacivi, Eva R. Stormanns, Mascha S. Recks, Stefanie Kuerten

## Notice of Republication

This article was republished on April 18, 2016, to correct text errors in the Methods, Results and Discussion sections that were introduced when an incorrect version was provided during the production process. Please download this article again to view the correct version. The originally published, uncorrected article and the republished, corrected article are provided here for reference.

## Supporting Information

S1 File Originally published, uncorrected article. (PDF)

S2 File Republished, corrected article. (PDF)
