# Supplementary material for: Correction: Time-Dependent Progression of Demyelination and Axonal Pathology in MP4-Induced Experimental Autoimmune Encephalomyelitis
Source: PLoS One. 2016 May 4;11(5):e0155197. doi: 10.1371/journal.pone.0155197 (PMC4856275; doi:10.1371/journal.pone.0155197)
Supplement: S2 File — (PDF) [file pone.0155197.s002.PDF]

PLOS ONE

RESEARCH ARTICLE

# Time-Dependent Progression of Demyelination and Axonal Pathology in MP4-Induced Experimental Autoimmune Encephalomyelitis

**Johanna Prinz[1☯], Aylin Karacivi[1☯], Eva R. Stormanns[1], Mascha S. Recks[2], Stefanie Kuerten[3]***

**1** Department of Anatomy I, University of Cologne, Joseph-Stelzmann-Str. 9, 50931, Cologne, Germany,
**2** Department of Anatomy II, University of Cologne, Joseph-Stelzmann-Str. 9, 50931, Cologne, Germany,
**3** Department of Anatomy and Cell Biology, University of Würzburg, Koellikerstraße 6, 97070, Würzburg, Germany

☯ These authors contributed equally to this work.
* stefanie.kuerten@uni-wuerzburg.de

CrossMark
click for updates

OPEN ACCESS

**Citation:** Prinz J, Karacivi A, Stormanns ER, Recks MS, Kuerten S (2015) Time-Dependent Progression of Demyelination and Axonal Pathology in MP4-Induced Experimental Autoimmune Encephalomyelitis. PLoS ONE 10(12): e0144847. doi:10.1371/journal.pone.0144847

**Editor:** Christoph Kleinschnitz, Julius-Maximilians-Universität Würzburg, GERMANY

**Received:** August 21, 2015

**Accepted:** November 24, 2015

**Published:** December 11, 2015

**Copyright:** © 2015 Prinz et al. This is an open access article distributed under the terms of the Creative Commons Attribution License, which permits unrestricted use, distribution, and reproduction in any medium, provided the original author and source are credited.

**Data Availability Statement:** All relevant data are within the paper and its Supporting Information files.

**Funding:** These authors have no support or funding to report.

**Competing Interests:** The authors have declared that no competing interests exist.

## Abstract

### Background

Multiple sclerosis (MS) is an autoimmune disease of the central nervous system (CNS) characterized by inflammation, demyelination and axonal pathology. Myelin basic protein/proteolipid protein (MBP-PLP) fusion protein MP4 is capable of inducing chronic experimental autoimmune encephalomyelitis (EAE) in susceptible mouse strains mirroring diverse histopathological and immunological hallmarks of MS. Limited availability of human tissue underscores the importance of animal models to study the pathology of MS.

### Methods

Twenty-two female C57BL/6 (B6) mice were immunized with MP4 and the clinical development of experimental autoimmune encephalomyelitis (EAE) was observed. Methylene blue-stained semi-thin and ultra-thin sections of the lumbar spinal cord were assessed at the peak of acute EAE, three months (chronic EAE) and six months after onset of EAE (long-term EAE). The extent of lesional area and inflammation were analyzed in semi-thin sections on a light microscopic level. The magnitude of demyelination and axonal damage were determined using electron microscopy. Emphasis was put on the ventrolateral tract (VLT) of the spinal cord.

### Results

B6 mice demonstrated increasing demyelination and severe axonal pathology in the course of MP4-induced EAE. In addition, mitochondrial swelling and a decrease in the nearest neighbor neurofilament distance (NNND) as early signs of axonal damage were evident with the onset of EAE. In semi-thin sections we observed the maximum of lesional area in

the chronic state of EAE while inflammation was found to a similar extent in acute and chronic EAE. In contrast to the well-established myelin oligodendrocyte glycoprotein (MOG) model, disease stages of MP4-induced EAE could not be distinguished by assessing the extent of parenchymal edema or the grade of inflammation.

## Conclusions

Our results complement our previous ultrastructural studies of B6 EAE models and suggest that B6 mice immunized with different antigens constitute useful instruments to study the diverse histopathological aspects of MS.

## Introduction

Multiple sclerosis (MS) is thought to be a chronic autoimmune disease of the central nervous system (CNS). For decades research has focused on identifying the mechanisms underlying demyelination and multifocal centers of inflammation in the white matter of the CNS [1–4]. Due to the limited availability of human CNS tissue animal models for MS are frequently used to perform mechanism-oriented studies [2, 5, 6]. One of the common models for MS is experimental autoimmune encephalomyelitis (EAE), in which susceptible animal strains are immunized with CNS antigens [7–10]. Originally, spinal cord homogenate (SCH) was used to induce EAE [11]. Today single proteins or peptides derived from those proteins in particular from the myelin sheath are typically employed. Among these proteins are myelin basic protein (MBP), proteolipid protein (PLP) or myelin oligodendrocyte glycoprotein (MOG) [1, 12–16]. None of the available EAE models can entirely mirror the immunopathology of MS on its own, but each model displays differential aspects of the disease entity. We have recently introduced a new model on the C57BL/6 (B6) background, the MBP-PLP fusion protein (MP4)-induced EAE, and we have distinguished several advantages of working with this antigen [16]. Among these advantages were the chronic disease course and the dependence on B cells and antibodies [17, 18]. Our studies have also shown fundamental differences in CNS histopathology when comparing MP4-induced EAE and the traditional MOG peptide 35–55 model with regard to the topography of lesions and the composition of CNS infiltrates [17, 19].

We have recently also initiated ultrastructural studies of MP4-induced EAE that were focused on the dorsal corticospinal tract (CST) and motor neuron damage. We have demonstrated that MP4-induced EAE was characterized by CST degeneration and alterations in the motor neuron perikaryal phosphorylation status [20]. There was an association between the clinical disease severity and the extent of CST degeneration [20]. These data were in parallel with results obtained in MS that demonstrated a relationship between the progression of neurodegeneration and disability [21, 22].

Our current study pays particular attention to the pathological changes in the ventrolateral tract (VLT) of the lumbar spinal cord. We investigated the morphological correlates of inflammation, demyelination and axonal damage studying both methylene blue-stained semi-thin sections and electron microscopic images. Our data show differential patterns of inflammation, demyelination and axonal damage in the course of MP4-induced EAE. In particular in comparison to both the chronic MOG:35–55 and the relapsing-remitting PLP:139–151 model that we have already characterized in detail, this study should provide valuable insights into the impact of immunization with different CNS antigens on the resulting CNS pathology.

# Materials and Methods

## Mice

Twenty-two six- to eight-week-old female wild-type (WT) B6 mice were obtained from Janvier Labs (Saint Berthevin Cedex, France) and kept under specific-pathogen conditions in the animal facilities of the Department of Anatomy of the University of Cologne, Germany. Mice were maintained in individually ventilated cages with autoclaved woodchip bedding in groups of two to five mice. Mice were fed a standard rodent diet (Altromin Spezialfutter GmbH & Co. KG, Lage, Germany) and had free access to pathogen-free water. From the time when mice displayed paralytic signs feed and water were offered at ground level. All animal experiments complied with the German Law on the Protection of Animals and the "Principles of laboratory animal care" (NIH publication No. 86–23, revised 1985) and the ARRIVE guidelines (S1 Appendix). The treatments were performed according to a protocol that was approved by the LANUV, Germany (approval number 2011.A276).

## Induction and assessment of EAE

MP4 was obtained from Alexion Pharmaceuticals (Cheshire, CT). Incomplete Freund's adjuvant (IFA) was prepared as a mixture of mannide monooleate (Sigma-Aldrich, St. Louis, MO) and paraffin oil (EM Science, Gibbstown, NJ), and complete Freund's adjuvant (CFA) was obtained by mixing *Mycobacterium tuberculosis* H37 Ra (Difco Laboratories, Franklin Lakes, NJ) at 5 mg/ml into IFA. For disease induction, mice were immunized subcutaneously in both sides of the flank with 200 μg MP4 (stock: 2 mg/ml) in CFA. Control mice remained untreated. On the day of immunization and 48 h later, 200 ng pertussis toxin (PTX; List Biological Laboratories, Hornby, ONT, Canada) were given in 500 μl sterile phosphate-buffered saline (PBS). The development of EAE was evaluated daily and the paralytic signs were classified following the standard scale: (0), no disease; (1), floppy tail; (2), hind limb weakness; (3), full hind limb paralysis; (4), quadriplegia; (5), death. Mice that were in between the clear-cut gradations of clinical signs were scored intermediate in increments of 0.5. Any mouse which was scored as a grade of 4 for more than 48 h was euthanized. Table 1 shows the classification of mice used in this study according to disease stage and EAE score.

## Analysis of methylene blue-stained spinal cord sections

For assessment of spinal cord histopathology, three transverse segments from the lumbar region were obtained from each mouse at the peak of acute paralytic signs (acute EAE), three months after onset of EAE (chronic EAE) and six months after onset of EAE (long-term EAE). Mice were sacrificed with $CO_2$ and perfused intracardially with 4% paraformaldehyde and 4% glutaraldehyde in 0.1M PBS (pH 7.4). Specimens were post-fixed at 4°C for at least 24 h before spinal cords were carefully removed from the vertebral canal. Samples were rinsed in 0.1M cacodylate buffer (pH 7.35) three times and fixed in 1% osmium on ice for 4 h. The tissue was then treated with 1.5% uranyl acetate in 70% ethanol for contrast enhancement and dehydrated in a graded series of ethanol at −20 C°. Sections were embedded in epon (Fluka, St. Louis, MO)

**Table 1. Number of mice in each group and mean value of the clinical score ± SD.**

| Disease stage | Time point of testing | Number of mice | EAE score ± SD |
|---|---|---|---|
| **Acute** | peak of acute EAE | 7 | 1.78 ± 0.75 |
| **Chronic** | three months after onset of EAE | 5 | 1.7 ± 0.57 |
| **Long-term** | six months after onset of EAE | 10 | 1.72 ± 0.53 |

doi:10.1371/journal.pone.0144847.t001

and polymerized at 60°C for at least three days. One μm thick semi-thin transverse sections were then cut on a Leica Ultracut UCT ultramicrotome (Leica Microsystems, Wetzlar, Germany) and stained with methylene blue. Ten to 25 images from 22 mice with two to four sections per mouse were evaluated using a light microscope (Leica DM LB2, Leica Microsystems, Wetzlar, Germany). Images were acquired using Zeiss AxioCam MRc and AxioVision 40 4.7 software (Carl Zeiss AG, Oberkochen, Germany). Subsequently, Image Pro Plus 6.0 software (Version 6.0, Media Cybernetics, Bethesda, MD, USA) was used to measure the size of the ventrolateral, the posterior and the corticospinal tract. Additionally, the percentage of the lesional area in each tract of the spinal cord was measured. The extent of parenchymal inflammation was assessed according to a semi-quantitative scale ranging from 0 (no cellular infiltration) to 5 (extensive cellular infiltration). The grade of edema was classified simultaneously using a semi-quantitative scale ranging from 0 (no edema) to 6 (extensive edema).

## Electron microscopic assessment

The evaluation of 80 nm thick ultra-thin sections was performed on a Zeiss EM 902 transmission electron microscope (Carl Zeiss AG, Oberkochen, Germany) and images were captured from the center of the lesional area in the VLT with a digital EM camera (MegaView III, Olympus Soft Imaging Systems GmbH, Münster, Germany). For the examination of acute EAE 21 sections of seven mice were investigated. In addition, 16 sections of five mice in the chronic stage and 23 sections of 10 mice with long-term EAE were analyzed. Seven images at 7000× magnification were acquired from each spinal cord section and the total number of nerve fibers/mm$^2$ was counted. Histological examination of myelin and axonal pathology was done blinded as to the clinical score and disease stage of the animals.

**Myelin pathology.** Image-Pro Plus software was used to count the number of normal appearing, completely demyelinated, or remyelinated nerve fibers, respectively, in addition to those in the process of demyelination. The *g-ratio* was used for the distinction between the different categories [23, 24], and calculated by dividing the diameter of the axon by the diameter of the whole nerve fiber. The optimal *g-ratio* for the ventrolateral column was set between 0.72 and 0.81 [23]. Axons showing a *g-ratio* below this level were characterized as *demyelinating*. Axons showing a *g-ratio* above this level were characterized as *remyelinating*. The term *physiological* was applied to axons with an intact myelin sheath and no other signs of axonal damage.

**Axonal damage.** Analysis of axonal damage comprised the assessment of complete axolysis, a decrease of the nearest neighbor neurofilament distance (NNND) [25] and mitochondrial swelling [26, 27]. The size of all mitochondria within an axon was set in relation to the total area of each axoplasm (*mito-ratio*). The cut-off value for mitochondrial swelling was set at 0.149 following our previously established analyses [28]. To achieve an objective evaluation of the NNND, the distance between the single neurofilaments was measured and the cut-off value for a decrease in NNND was set at 0.039 μm [28]. While complete degeneration of axons (axolysis) was defined as *gross* axonal pathology, mitochondrial swelling and a NNND decrease were designated as *fine* axonal damage [25, 27, 29]. The extent of myelin and axonal pathology as described above was assessed for each nerve fiber and the number of fibers with isolated myelin or axonal damage or combined pathology was evaluated.

## Statistical analysis

SigmaPlot software (Version 12.0, Systat Software, San Jose, CA, USA) was used for statistical analysis. In case of a normal distribution, differences between groups were assessed using Student`s t-test. The Mann-Whitney U rank-sum test was applied in case the normality test failed. Three levels of statistical significance were differentiated: $^{*}p \leq 0.05$, $^{**}p < 0.01$ and

***p < 0.001. Spearman`s correlation was used to assess the correlation between axolysis and mitochondrial damage or axolysis and a decreased NNND, respectively.

## Results

### Inflammation and neurodegeneration coexist in the course of MP4-induced EAE

Fig 1 delineates the localization of the VLT in a lumbar section of the murine spinal cord. A semi-quantitative scoring system as described in *Materials and methods* was used to analyze the dimension of inflammation and edema in MP4-induced EAE. The size of the lesional area was determined as a morphological correlate of neurodegeneration. The mean pathological score of inflammation and the mean value of the spinal cord lesional area expressed as percentage are shown in Fig 2A and 2E and representative images are demonstrated in Fig 2B–2D and 2F–2H. Inflammation was found to a similar extent in the acute and chronic stage of EAE, while it decreased in the long-term stage of the disease. Of note, in contrast to the MOG:35–55 model [28, 30] the spinal cord of B6 mice immunized with MP4 hardly showed any parenchymal edema, while its extent was still slightly above the level of the control mice (data not shown). The lesional area significantly increased from acute to chronic EAE and again decreased in the long-term stage of EAE.

### Progressive myelin pathology is associated with the disease kinetics in MP4-induced EAE

In the context of an autoimmune disease such as MS, several self-antigens are affected. In MS and EAE these targets typically belong to the myelin sheath and include MBP, MOG, PLP, myelin-associated glycoprotein (MAG) or neuronal structures [31]. Nerve fibers being in the process of demyelination morphologically differ from physiological axons. The myelin sheath

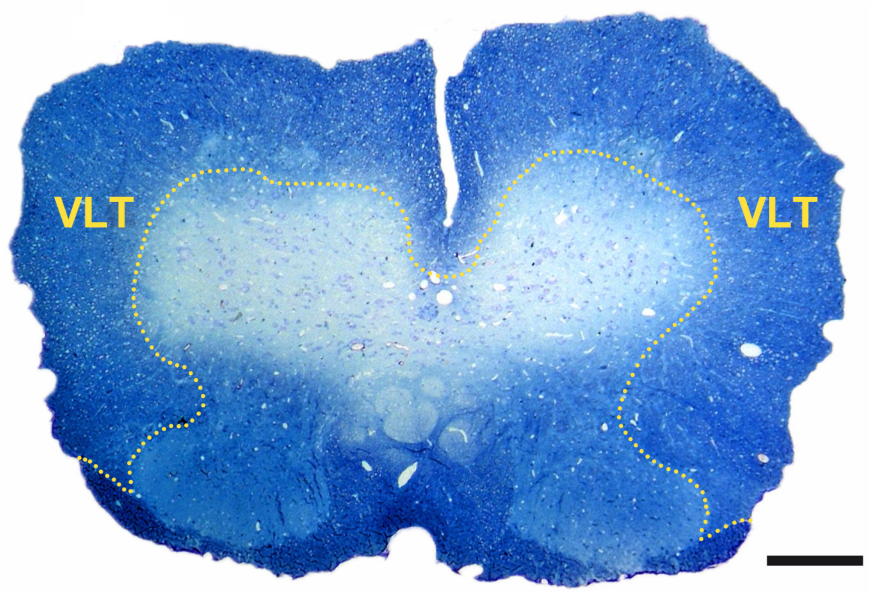


**Fig 1. Localization of the VLT in the murine spinal cord.** The image shows a methylene blue-stained transverse section of murine lumbar spinal cord in a MP4-immunized mouse in the long-term stage of the disease. The VLT is circled. The scale bar depicts 200 μm.

doi:10.1371/journal.pone.0144847.g001

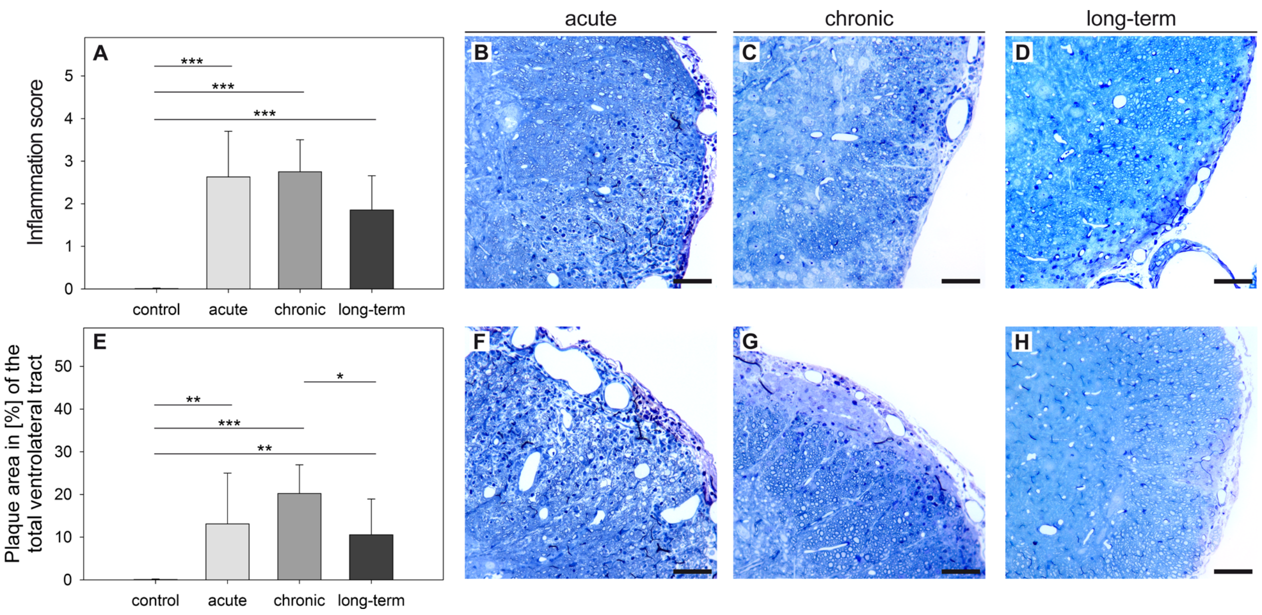


**Fig 2. Time-dependent alterations of inflammation and lesional area in MP4-induced EAE.** Semi-thin sections of the VLT were obtained from B6 mice in acute EAE, three months (chronic) and six months after EAE onset (long-term). Sections were stained with methylene blue and compared to a non-immunized control group that consisted of $n = 10$ mice. Cellular infiltration (A) was graded on a semi-quantitative scale and the lesional area size was measured (E). The means ± SD are shown for a total number of $n = 7$ mice in the acute, $n = 5$ mice in the chronic and $n = 10$ mice in the long-term stage of EAE. Representative images from every stage of the disease are displayed showing cellular inflammation (B-D) and the lesional area (F-H). $^{*}p \leq 0.05$; $^{**}p \leq 0.01$; $^{***}p \leq 0.001$. Scale bars depict 50 μm.

doi:10.1371/journal.pone.0144847.g002

shows swelling and myelin lamellae begin to diverge widely from each other [32, 33]. Representative images are shown in Fig 3. We evaluated ultrastructural images of the VLT at 7000× magnification and classified each axon as being in the process of demyelination or completely demyelinated. For this purpose we assessed the *g-ratio* as described in *Materials and methods*. The following aspects were remarkable. On the one hand, we found a high number of axons in the process of demyelination in the acute stage and this number further increased slightly in chronic EAE (Fig 3A–3E). In long-term EAE significantly less demyelinating fibers were observed (Fig 3A–3E). In contrast, complete degeneration of the myelin sheath was evident throughout the course of the disease and reached a maximum in the long-term stage (Fig 3F–3J). Axons displaying isolated myelin damage (i.e. without any signs of axonal pathology) were already evident in the acute stage of EAE and their numbers significantly increased with the progression of the disease (acute 568,691.42 ± 421,727.94 per $mm^2$, chronic 888,207 ± 318,821.72 per $mm^2$ and long-term 1,495,870.6 ± 764,138.56 per $mm^2$, with acute *vs* chronic: $p = 0.164$, chronic *vs* long-term: $p = 0.067$ and acute *vs* long-term: $p = 0.027$).

## There was no evidence of spinal cord remyelination associated with MP4-induced EAE

Remyelinated nerve fibers are characterized by dense and thin myelin lamellae [34]. We evaluated ultrastructural images of the VLT at 7000× magnification and detected nerve fibers that were in the state of regeneration (Fig 3K and 3L). The data show that both demyelination and remyelination coexisted at the same time. The occurrence of remyelinated nerve fibers was not just restricted to the long-term stage of EAE, but was also present in the acute and chronic state of the disease. However, the extent of remyelinating nerve fibers was below the level that was evident in non-immunized control mice. Expressed as a percentage we only found 0.21% ±

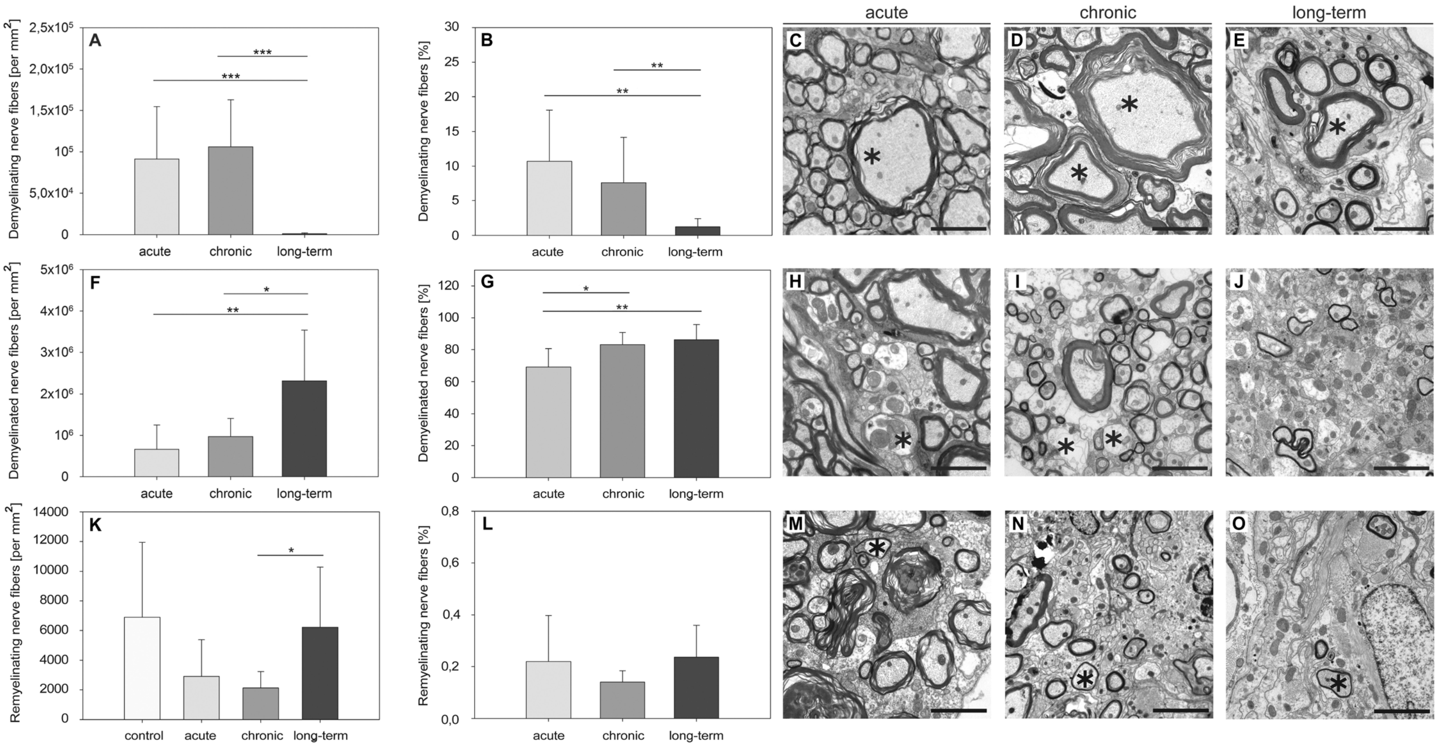


**Fig 3. Differences in myelin pathology in the course of MP4-induced EAE.** Ultra-thin spinal cord sections were obtained from MP4-immunized B6 mice in different disease stages and images were taken from the center of the lesional area in the VLT. The numbers and percentages of nerve fibers being either in the process of demyelination (A, B), completely demyelinated (F, G) or remyelinated (K, L) were evaluated. Values obtained from non-immunized control mice were subtracted for the nerve fibers being in the process of demyelination and for completely demyelinated nerve fibers. The means ± SD are demonstrated for a total number of $n = 7$ control mice, $n = 7$ mice in the acute, $n = 5$ mice in the chronic and $n = 10$ mice in the long-term stage of the disease. Representative images from every stage of EAE depicting demyelinating (C-E), demyelinated (H-J) or remyelinating nerve fibers (M-O) are displayed. $*p \leq 0.05$; $**p \leq 0.01$; $***p \leq 0.001$. Scale bars depict 10 μm.

doi:10.1371/journal.pone.0144847.g003

0.17% remyelinating nerve fibers in the acute, 0.14% ± 0.04% in the chronic and 0.23% ± 0.12% in the long-term stage of EAE. Fig 3M–3O shows representative images of remyelinating nerve fibers in every stage of the disease.

## The maximum of axonal damage was observed in long-term EAE

We examined the axoplasm at 7000× magnification putting emphasis on axolysis, mitochondrial swelling and a decrease in the NNND as morphological correlates for axonal damage [25, 26]. Mitochondrial swelling in relation to the total axonal area was measured and the *mito-ratio* was determined. A significant number of axons with an increased *mito-ratio* was observed in each stage of EAE (Fig 4A–4E). Next, we analyzed the NNND as described in *Materials and methods* and measured the distance between the single neurofilaments in μm whose decrease is considered to be an early sign of neurodegeneration [25]. A significant number of axons with a decreased NNND was evident in the course of EAE (Fig 4F–4J). In addition, we examined the amount of axolysis as a correlate of irreversible axonal damage. Fig 4K–4O show that axolysis already existed in an early stage of MP4-induced EAE and significantly increased with disease progression. There was a significant correlation between axolysis and a decrease in the NNND ($r_s = 0.660$ and $p = 0.0008$) or axolysis and mitochondrial swelling, respectively ($r_s = 0.519$ and $p = 0.0135$, Fig 5A and 5B). Isolated axonal damage (i.e. without evidence of myelin pathology) was already observed in the early stage of MP4-induced EAE and significantly increased in

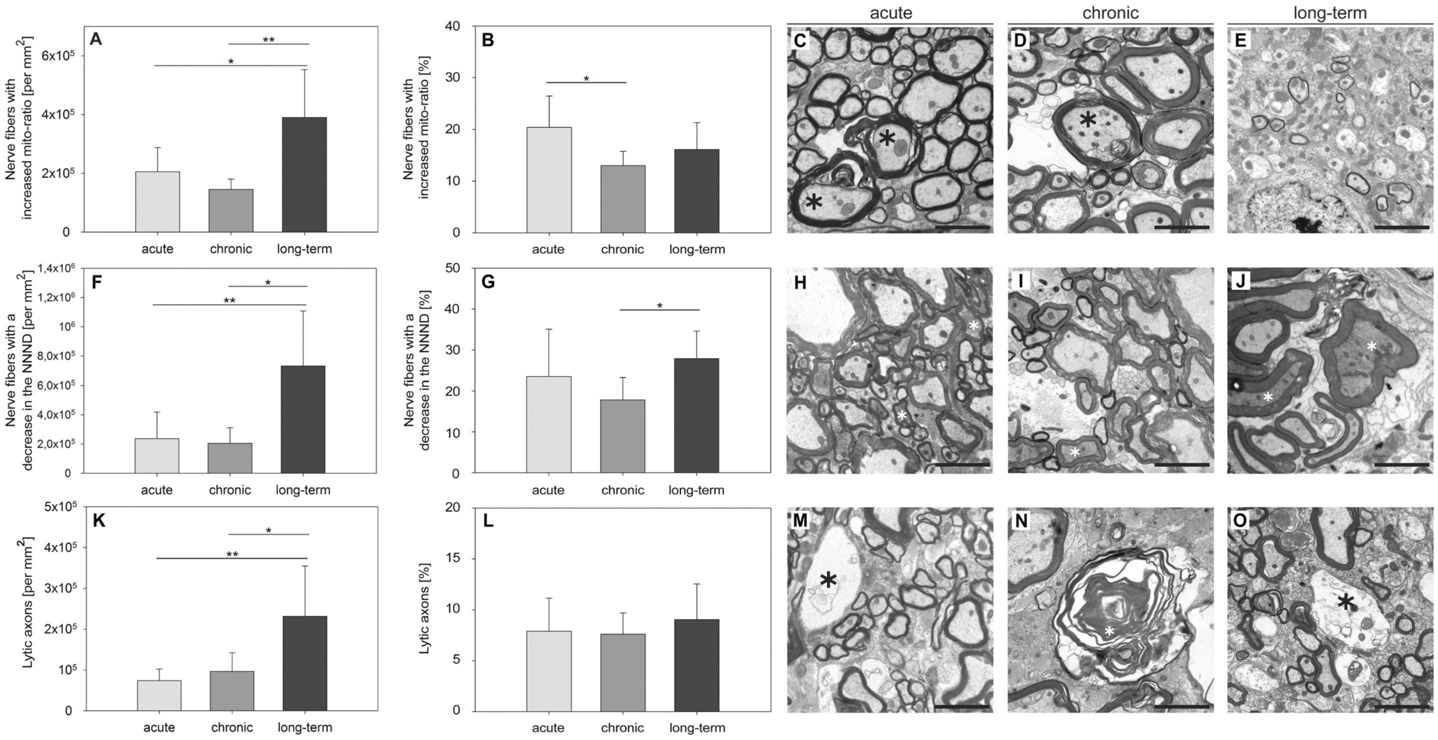


**Fig 4. The extent of axonal pathology peaks six months after the onset of EAE in MP4-immunized B6 mice.** Ultra-thin spinal cord sections were obtained and images were taken from the center of the lesional area in the VLT. The numbers and percentages of axons showing mitochondrial swelling (A, B), a decreased NNND (F, G) or complete axolysis (K, L) were evaluated. Values obtained from non-immunized control mice were subtracted from the absolute number. The means ± SD are demonstrated for a total of $n = 7$ mice with acute, $n = 5$ mice with chronic and $n = 10$ mice with long-term EAE. Representative images obtained in each disease stage are providing examples of mitochondrial swelling (C-E), a decrease in the NNND (H-J) and axolysis (M-O). *$p \leq 0.05$; **$p \leq 0.01$; ***$p \leq 0.001$. Scale bars depict 10 μm.

doi:10.1371/journal.pone.0144847.g004

long-term disease (acute 71,747.42 ± 34,588.28 per mm$^2$, chronic 27,529.8 ± 11,802.62 per mm$^2$ and long-term 121,344.9 ± 33,316.92 per mm$^2$, with acute *vs* long-term: $p = 0.009$ and chronic *vs* long-term: $p < 0.001$). The combination of both myelin and axonal pathology first

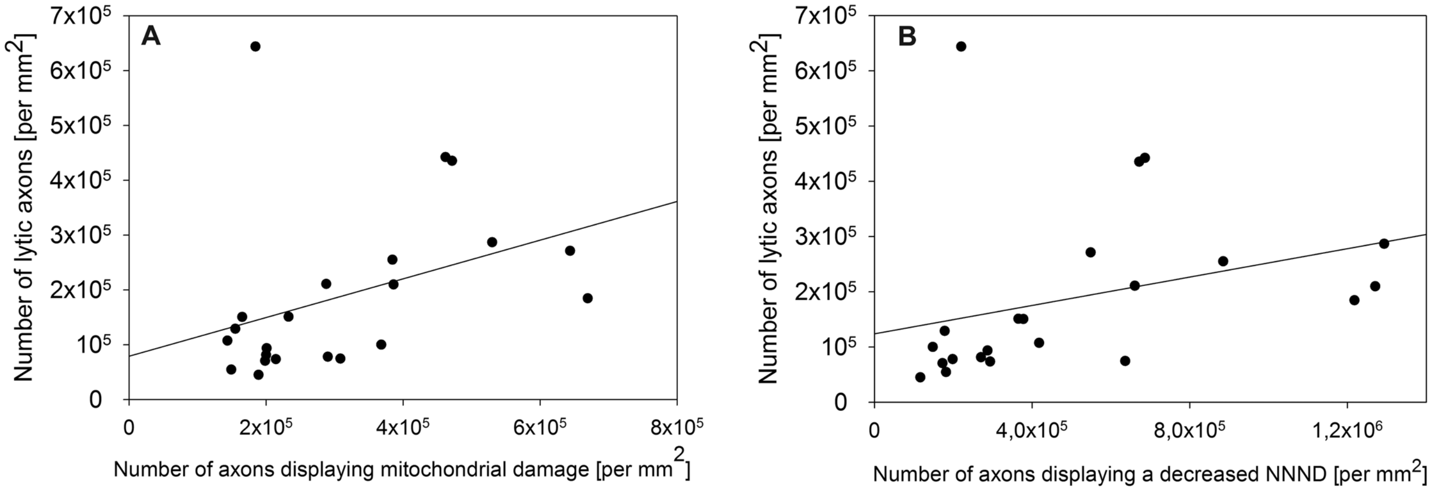


**Fig 5. Correlation between axolysis and mitochondrial swelling or axolysis and a decreased NNND, respectively.** (A) Significant correlation between the number of lytic axons and the number of axons showing mitochondrial swelling ($r_s = 0.519$ and $p = 0.0135$). (B) Significant correlation between the number of lytic axons and the number of axons with a decreased NNND ($r_s = 0.660$ and $p = 0.000792$). Results refer to $n = 22$ MP4-immunized mice.

doi:10.1371/journal.pone.0144847.g005

reached a plateau within the first three months of EAE before it further significantly increased six months after the onset of the disease (acute 465,760.42 ± 206,287.55 per $mm^2$, chronic 468,415 ± 142,799.45 per $mm^2$ and long-term 1,082,142.7 ± 530,122.17 per $mm^2$, with acute *vs* long-term: $p = 0.011$ and chronic *vs* long-term: $p = 0.027$). In relation to the total number of axons we found 47.5% ± 12.4% isolated myelin damage in the acute, 63.2% ± 7.2% in the chronic and 53.6% ± 8.3% in the long-term stage of EAE. Isolated axonal damage was observed to a lower extent in the acute 8.0% ± 5.5% and chronic stage 2.3% ± 1.7% and six months after the onset of EAE 6.4% ± 5.1%. In the acute stage, 44.5% ± 12.5% nerve fibers showed the combination of both myelin and axonal damage compared to 34.5% ± 6.6% in the chronic and 40% ± 6.6% in the long-term stage of the disease.

## Discussion

MS is characterized by inflammation, demyelination and axonal damage [3, 4, 35]. In particular autoreactive T cells, activated microglia and macrophages are thought to constitute a major part of the inflammatory component [36, 37]. Only recently, however, B cell aggregates were found in the meninges of patients with secondary progressive MS and associated with more severe cortical pathology [38, 39]. The limited availability of human CNS tissue makes it necessary to focus research on suitable animal models in order to get further insights into the pathology underlying irreversible functional deficits in patients. One of the most commonly used animal models is EAE, which is elicited by immunization of susceptible mouse strains with CNS antigens or by the passive transfer of encephalitogenic T cells [2]. There are also spontaneous models that rely on genetic manipulations of the T cell receptor [40]. The genetic background of the animal strain and the antigens used for disease induction have an impact on the clinical course of the disease and the histopathological features of each model [41].

In our previous studies we compared the clinical outcome, cellular composition of CNS infiltrates and features of neurodegeneration in B6 mice immunized with PLP:178–191, MOG:35–55 or MP4 [16, 17, 42]. PLP:178-191-elicited EAE was characterized by a delayed EAE onset and showed a chronic disease course [19]. Further studies of this model showed a loss of axonal density in the VLT in the absence of pathological alterations of the dorsal tract or the motor neurons. Additionally, we did not observe substantial demyelination in the course of the disease [42]. B6 mice immunized with other PLP peptides such as PLP:43–64, PLP:50–64 or PLP:94–108 did not display demyelination either [43]. Therefore and because of the difficulty of working with the whole PLP protein, studies that rely on PLP-induced EAE rather play a minor role in our current understanding of MS pathogenesis. In contrast, MOG:35–55 is one of the most frequently used EAE antigens that causes chronic disease, extensive spinal cord inflammation and demyelination in B6 mice [13, 44]. While inflammation could be found only in early stages of MOG:35-55-induced EAE, myelin damage was consistent throughout the disease, but significantly decreased with disease progression [28]. No significant differences in the composition of cellular infiltration could be found comparing the acute and chronic stage of MOG:35-55-induced EAE [17]. Our previous thorough histological characterization of PLP:178-191- and MOG:35-55-induced EAE on the B6 background enabled us to make direct comparisons with MP4-immunized EAE in the current study.

Our previous research has provided clear evidence that MP4-induced EAE is B cell-dependent, not only because B cell-deficient mice were resistant to EAE induction [16], but also a remarkable amount of B cells was found within the CNS infiltrates, which formed B cell aggregates and tertiary lymphoid organs as disease progressed [45]. Evidence that B cells are crucially involved in the pathogenesis of MS is accumulating and has lead to the implementation of new B cell-specific therapies. The monoclonal chimeric antibody rituximab (Mabthera®) is

directed against the B cell CD20 antigen and was originally approved for the treatment of non-Hodgkin`s lymphoma (NHL) in 1997 [46]. In patients with MS rituximab induces a long-lasting depletion of the $CD20^+$ B cells. Hauser et al. demonstrated that patients with relapsing-remitting MS (RRMS) treated with rituximab showed a reduced number of gadolinium-enhancing lesions as detected by magnetic resonance imaging (MRI) scans of the brain [47]. The monoclonal anti-CD52 antibody alemtuzumab (MabCampath®) depletes CD52 expressing B cells, T cells, monocytes and macrophages without affecting stem cells [48]. Comparing interferon-beta 1a treatment of RRMS to alemtuzumab over two years showed that only 22% of patients treated with alemtuzumab developed a relapse compared to nearly 60% of the patients treated with interferon-beta 1a. [49]. Another novel monoclonal antibody for the treatment of MS is the humanized anti-CD20 antibody ocrelizumab [50]. Patients receiving ocrelizumab in a phase II trial had up to 96% lower gadolinium-enhancing MRI lesions and relapse rates were up to 80% lower than in the placebo and an interferon-beta 1a group [51]. Ocrelizumab will probably be approved for the treatment of MS in the beginning of 2016.

In contrast to the MOG:35–55 model MP4 triggered an initial wave of immune cell infiltration into the brain, which disappeared in the course of EAE and shifted to the cerebellar white matter [19]. Therefore, not only the comparison of different EAE models, but also of different CNS regions allowed conclusions as to distinct patterns of inflammation, demyelination and nerve fiber damage [20]. Studying the CST of MP4-immunized B6 mice we found not only axonal damage and myelin degeneration, but also identified differences in the degree of motor neuron perikaryal phosphorylation as a morphological correlate for gray matter damage [20]. In the same model we observed severe nuclear membrane defects and a decrease in the quantity of synapses in addition to a beginning disintegration of the rough endoplasmatic reticulum [20]. In our present study we focused our analysis on the VLT accounting for the largest part of the murine spinal cord. So far, previous studies on MP4-induced EAE have not dealt with an ultrastructural characterization and it was our aim to delineate whether this model was a valuable tool for ultrastructural studies of MS histopathology. The assessment of semi-thin sections demonstrated that similar to the MOG:35–55 model inflammation was present within the first three months after the onset of the disease. However, in contrast to the MOG:35–55 model, which showed a continuous decrease of inflammation with disease progression [30], we observed that inflammation was nearly in a steady state in the course of MP4-induced EAE. Another remarkable difference between the two animal models was the extent of the parenchymal edema. Analyzing the VLT of the MP4 model, hardly any edema could be detected, while in the MOG:35–55 model marked edema was observed at least in the beginning of the EAE [30]. The analysis of regeneration demonstrated a significant increase in the number of remyelinated nerve fibers in the MOG:35–55 model, in particular in the long-term stage of EAE. Furthermore, remyelination was found to a greater extent in the VLT than in the dorsolateral tract of the spinal cord and was significantly increased compared to non-immunized control mice [28]. In MP4-induced EAE the numbers of remyelinating nerve fibers were below the numbers measured in non-immunized control mice, which suggests that this model is characterized by irreversible spinal cord damage. Comparing the MOG:35–55 and the MP4 model in regard to spinal cord regeneration might be a suitable tool to study mechanisms of neurodegeneration and–regeneration and treatment options addressing this aspect of MS pathology.

In MP4-induced EAE both demyelination and axolysis could be detected with the onset of the disease. These data are in line with our previous ultrastructural studies of the MOG:35–55 model that showed axonal pathology in the VLT from the earliest disease stages on [28]. Similarly, analyses of biopsy and autopsy tissues from the CNS of patients demonstrated demyelination and axonal transection in early disease stages [4, 21]. Our results also show that the numbers of completely demyelinated axons increased in the course of the disease.

Simultaneously with this increase in demyelinated nerve fibers the numbers of lytic axons increased significantly underlining the traditional notion that axonal injury is the result of demyelination [4]. Furthermore, we observed an elevated number of axons with a decreased NNND. A decrease in the NNND, which depends on the phosphorylation status of neurofilament sidearms, is considered to coincide with a loss of myelination [21, 25, 52, 53]. Research on *post mortem* human CNS tissue showed an increase of dephosphorylated neurofilaments in MS patients while a high amount of hyperphosphorylated neurofilaments could be detected in acute lesions [53], representing the changing dynamics of axonal pathology in the course of MS. Ultrastructural examinations of the CST and motor neurons in B6 mice immunized with MOG:35–55 or MP4, respectively, demonstrated a decrease in the NNND in both models. Another feature of axonal pathology that we investigated in the present study was the extent of mitochondrial swelling. Here we show that there was no significant degree of mitochondrial swelling in the beginning of EAE, while it was evident with the transition to chronic disease. In contrast to this finding mitochondrial swelling could be detected early on in the MOG:35–55 model [28]. Both features, a decrease in the NNND and mitochondrial swelling are known to be early signs of axonal damage [25–27]. Accordingly, we found a significant correlation between axolysis and both a decreased NNND and mitochondrial swelling.

Another well-established animal model is the non-obese diabetic (NOD) mouse that develops spontaneous diabetes. This mouse strain was formerly developed to establish a cataract-prone subline (CTS) and it is the most common model for investigating the human autoimmune type I diabetes [54–56]. Immunization of NOD mice with MOG:35–55 results in an initial acute EAE attack, followed by a progressively worsening relapse mirroring secondary progressive MS (SPMS) [57]. Mayo et al. have created a hybrid mouse strain by cross-breeding NOD and B6 mice. These mice showed chronic progressive EAE after immunization with MOG:35–55 [58]. The introduction of a MP4/NOD model may be an additional valuable tool to further investigate the histopathological hallmarks of progressive MS.

## Supporting Information

**S1 Appendix. ARRIVE guidelines.**
(PDF)

## Acknowledgments

We would like to thank Daniel Behr and Emanuel Klimschak for valuable support. The authors received no specific funding for this work.

## Author Contributions

Conceived and designed the experiments: JP AK MR SK. Performed the experiments: JP AK ES SK. Analyzed the data: JP AK SK. Wrote the paper: JP SK.

## References

1. Sospedra M, Martin R. Immunology of multiple sclerosis*. Annual Review of Immunology. 2005; 23 (1):683–747. doi: 10.1146/annurev.immunol.23.021704.115707
2. Gold R. Understanding pathogenesis and therapy of multiple sclerosis via animal models: 70 years of merits and culprits in experimental autoimmune encephalomyelitis research. Brain. 2006; 129 (8):1953–71. doi: 10.1093/brain/awl075
3. Lassmann H, Bruck W, Lucchinetti CF. The immunopathology of multiple sclerosis: An overview. Brain pathology. 2007; 17(2):210–8. doi: 10.1111/j.1750-3639.2007.00064.x PMID: 17388952.

4. Bitsch A, Schuchardt J, Bunkowski S, Kuhlmann T, Bruck W. Acute axonal injury in multiple sclerosis. Correlation with demyelination and inflammation. Brain. 2000; 123 (Pt 6):1174–83. PMID: 10825356.
5. Goverman J, Brabb T. Rodent models of experimental allergic encephalomyelitis applied to the study of multiple sclerosis. Laboratory animal science. 1996; 46(5):482–92. PMID: 8905579.
6. Zamvil SS, Mitchell DJ, Moore AC, Kitamura K, Steinman L, Rothbard JB. T-cell epitope of the autoantigen myelin basic protein that induces encephalomyelitis. Nature. 1986; 324(6094):258–60. doi: 10.1038/324258a0 PMID: 2431317.
7. Schmidt S. Candidate autoantigens in multiple sclerosis. Multiple Sclerosis. 1999; 5(3):147–60. doi: 10.1177/135245859900500303 PMID: 10408714
8. Wekerle H, Kojima K, Lannes-Vieira J, Lassmann H, Linington C. Animal models. Ann Neurol. 1994; 36 Suppl:S47–53. PMID: 7517126.
9. Steinman L. Assessment of animal models for ms and demyelinating disease in the design of rational therapy. Neuron. 1999; 24(3):511–4. PMID: 10595504.
10. Handel AE, Lincoln MR, Ramagopalan SV. Of mice and men: Experimental autoimmune encephalitis and multiple sclerosis. European Journal of Clinical Investigation. 2011; 41(11):1254–8. doi: 10.1111/j.1365-2362.2011.02519.x PMID: 21418205
11. Bernard CC. Experimental autoimmune encephalomyelitis in mice: Genetic control of susceptibility. Journal of immunogenetics. 1976; 3(4):263–74. PMID: 1109134.
12. Bernard CC, Johns TG, Slavin A, Ichikawa M, Ewing C, Liu J, et al. Myelin oligodendrocyte glycoprotein: A novel candidate autoantigen in multiple sclerosis. Journal of molecular medicine. 1997; 75 (2):77–88. PMID: 9083925.
13. Mendel I, Kerlero De Rosbo N, Ben-Nun A. A myelin oligodendrocyte glycoprotein peptide induces typical chronic experimental autoimmune encephalomyelitis in h-2b mice: Fine specificity and t cell receptor v[beta] expression of encephalitogenic t cells. European journal of immunology. 1995; 25(7):1951–9. doi: 10.1002/eji.1830250723 PMID: 7621871.
14. Oliver AR, Lyon GM, Ruddle NH. Rat and human myelin oligodendrocyte glycoproteins induce experimental autoimmune encephalomyelitis by different mechanisms in c57bl/6 mice. The Journal of Immunology. 2003; 171(1):462–8. doi: 10.4049/jimmunol.171.1.462 PMID: 12817031
15. Tompkins SM, Padilla J, Dal Canto MC, Ting JP-Y, Van Kaer L, Miller SD. De novo central nervous system processing of myelin antigen is required for the initiation of experimental autoimmune encephalomyelitis. The Journal of Immunology. 2002; 168(8):4173–83. doi: 10.4049/jimmunol.168.8.4173 PMID: 11937578
16. Kuerten S, Lichtenegger FS, Faas S, Angelov DN, Tary-Lehmann M, Lehmann PV. Mbp-plp fusion protein-induced eae in c57bl/6 mice. Journal of Neuroimmunology. 2006; 177(1–2):99–111. doi: 10.1016/j.jneuroim.2006.03.021 PMID: 16781782
17. Kuerten S, Javeri S, Tary-Lehmann M, Lehmann PV, Angelov DN. Fundamental differences in the dynamics of cns lesion development and composition in mp4- and mog peptide 35–55-induced experimental autoimmune encephalomyelitis. Clinical Immunology. 2008; 129(2):256–67. doi: 10.1016/j.clim.2008.07.016 PMID: 18722816
18. Kuerten S, Pauly R, Blaschke S, Rottlaender A, Kaiser CC, Schroeter M, et al. [the significance of a b cell-dependent immunopathology in multiple sclerosis]. Fortschritte der Neurologie-Psychiatrie. 2011; 79(2):83–91. doi: 10.1055/s-0029-1245937 PMID: 21253995.
19. Kuerten S, Kostovabales D, Frenzel L, Tigno J, Tarylehmann M, Angelov D, et al. Mp4- and mog:35–55-induced eae in c57bl/6 mice differentially targets brain, spinal cord and cerebellum☆. Journal of Neuroimmunology. 2007; 189(1–2):31–40. doi: 10.1016/j.jneuroim.2007.06.016 PMID: 17655940
20. Gruppe TL, Recks MS, Addicks K, Kuerten S. The extent of ultrastructural spinal cord pathology reflects disease severity in experimental autoimmune encephalomyelitis. Histology and histopathology. 2012; 27(9):1163–74. PMID: 22806903.
21. Trapp BD, Peterson J, Ransohoff RM, Rudick R, Mork S, Bo L. Axonal transection in the lesions of multiple sclerosis. The New England journal of medicine. 1998; 338(5):278–85. doi: 10.1056/NEJM199801293380502 PMID: 9445407.
22. De Stefano N, Matthews PM, Fu L, Narayanan S, Stanley J, Francis GS, et al. Axonal damage correlates with disability in patients with relapsing-remitting multiple sclerosis. Results of a longitudinal magnetic resonance spectroscopy study. Brain. 1998; 121 (Pt 8):1469–77. PMID: 9712009.
23. Chomiak T, Hu B. What is the optimal value of the g-ratio for myelinated fibers in the rat cns? A theoretical approach. PloS one. 2009; 4(11):e7754. doi: 10.1371/journal.pone.0007754 PMID: 19915661; PubMed Central PMCID: PMC2771903.
24. Guy J, Ellis EA, Hope GM, Emerson S. Maintenance of myelinated fibre g ratio in acute experimental allergic encephalomyelitis. Brain. 1991; 114 (Pt 1A):281–94. PMID: 1998887.

25. Lunn MP, Crawford TO, Hughes RA, Griffin JW, Sheikh KA. Anti-myelin-associated glycoprotein antibodies alter neurofilament spacing. Brain. 2002; 125(Pt 4):904–11. PMID: 11912122.

26. Mao P, Reddy PH. Is multiple sclerosis a mitochondrial disease? Biochimica et biophysica acta. 2010; 1802(1):66–79. doi: 10.1016/j.bbadis.2009.07.002 PMID: 19607913; PubMed Central PMCID: PMC2790545.

27. Schon EA, Manfredi G. Neuronal degeneration and mitochondrial dysfunction. The Journal of clinical investigation. 2003; 111(3):303–12. doi: 10.1172/JCI17741 PMID: 12569152; PubMed Central PMCID: PMC151870.

28. Recks MS, Stormanns ER, Bader J, Arnhold S, Addicks K, Kuerten S. Early axonal damage and progressive myelin pathology define the kinetics of cns histopathology in a mouse model of multiple sclerosis. Clinical Immunology. 2013; 149(1):32–45. doi: 10.1016/j.clim.2013.06.004 PMID: 23899992

29. Mao P, Reddy PH. Is multiple sclerosis a mitochondrial disease? Biochimica et Biophysica Acta (BBA) —Molecular Basis of Disease. 2010; 1802(1):66–79. doi: 10.1016/j.bbadis.2009.07.002

30. Recks MS, Addicks K, Kuerten S. Spinal cord histopathology of mog peptide 35–55-induced experimental autoimmune encephalomyelitis is time- and score-dependent. Neuroscience Letters. 2011; 494 (3):227–31. doi: 10.1016/j.neulet.2011.03.021 PMID: 21406210

31. Recks MS, Bader J, Kaiser CC, Schroeter M, Fink GR, Addicks K, et al. [axonal damage and its significance for the concept of neurodegeneration in multiple sclerosis]. Fortschritte der Neurologie-Psychiatrie. 2011; 79(3):161–70. doi: 10.1055/s-0029-1246014 PMID: 21394707.

32. Dyer CA. The structure and function of myelin: From inert membrane to perfusion pump. Neurochemical research. 2002; 27(11):1279–92. PMID: 12512934.

33. Merrill JE, Scolding NJ. Mechanisms of damage to myelin and oligodendrocytes and their relevance to disease. Neuropathology and applied neurobiology. 1999; 25(6):435–58. PMID: 10632895.

34. Bondan EF, Lallo MA, Graca DL. Ultrastructural study of the effects of cyclosporine in the brainstem of wistar rats submitted to the ethidium bromide demyelinating model. Arquivos de neuro-psiquiatria. 2008; 66(2B):378–84. PMID: 18641876.

35. Calabrese M, Filippi M, Gallo P. Cortical lesions in multiple sclerosis. Nature reviews Neurology. 2010; 6(8):438–44. doi: 10.1038/nrneurol.2010.93 PMID: 20625376.

36. Aloisi F, Ria F, Adorini L. Regulation of t-cell responses by cns antigen-presenting cells: Different roles for microglia and astrocytes. Immunology today. 2000; 21(3):141–7. PMID: 10689302.

37. Neumann H, Medana IM, Bauer J, Lassmann H. Cytotoxic t lymphocytes in autoimmune and degenerative cns diseases. Trends in neurosciences. 2002; 25(6):313–9. PMID: 12086750.

38. Howell OW, Reeves CA, Nicholas R, Carassiti D, Radotra B, Gentleman SM, et al. Meningeal inflammation is widespread and linked to cortical pathology in multiple sclerosis. Brain. 2011; 134(Pt 9):2755–71. doi: 10.1093/brain/awr182 PMID: 21840891.

39. Magliozzi R, Howell O, Vora A, Serafini B, Nicholas R, Puopolo M, et al. Meningeal b-cell follicles in secondary progressive multiple sclerosis associate with early onset of disease and severe cortical pathology. Brain. 2007; 130(Pt 4):1089–104. doi: 10.1093/brain/awm038 PMID: 17438020.

40. Pollinger B, Krishnamoorthy G, Berer K, Lassmann H, Bosl MR, Dunn R, et al. Spontaneous relapsing-remitting eae in the sjl/j mouse: Mog-reactive transgenic t cells recruit endogenous mog-specific b cells. The Journal of experimental medicine. 2009; 206(6):1303–16. doi: 10.1084/jem.20090299 PMID: 19487416; PubMed Central PMCID: PMC2715069.

41. Lassmann H. Comparative neuropathology of chronic experimental allergic encephalomyelitis and multiple sclerosis. Schriftenreihe Neurologie. 1983; 25:1–135. PMID: 6605578.

42. Kuerten S, Gruppe TL, Laurentius L-M, Kirch C, Tary-Lehmann M, Lehmann PV, et al. Differential patterns of spinal cord pathology induced by mp4, mog peptide 35–55, and plp peptide 178–191 in c57bl/6 mice. APMIS. 2011; 119(6):336–46. doi: 10.1111/j.1600-0463.2011.02744.x PMID: 21569091

43. Kuerten S, Angelov DN. Comparing the cns morphology and immunobiology of different eae models in c57bl/6 mice—a step towards understanding the complexity of multiple sclerosis. Annals of anatomy = Anatomischer Anzeiger: official organ of the Anatomische Gesellschaft. 2008; 190(1):1–15. doi: 10.1016/j.aanat.2007.11.001 PMID: 18342137.

44. Clements CS, Reid HH, Beddoe T, Tynan FE, Perugini MA, Johns TG, et al. The crystal structure of myelin oligodendrocyte glycoprotein, a key autoantigen in multiple sclerosis. Proceedings of the National Academy of Sciences of the United States of America. 2003; 100(19):11059–64. doi: 10.1073/pnas.1833158100 PMID: 12960396; PubMed Central PMCID: PMC196926.

45. Kuerten S, Schickel A, Kerkloh C, Recks MS, Addicks K, Ruddle NH, et al. Tertiary lymphoid organ development coincides with determinant spreading of the myelin-specific t cell response. Acta Neuropathol. 2012; 124(6):861–73. doi: 10.1007/s00401-012-1023-3 PMID: 22842876.

46. Dotan E, Aggarwal C, Smith MR. Impact of rituximab (rituxan) on the treatment of b-cell non-hodgkin's lymphoma. P & T: a peer-reviewed journal for formulary management. 2010; 35(3):148–57. PMID: 20442809; PubMed Central PMCID: PMC2844047.

47. Hauser SL, Waubant E, Arnold DL, Vollmer T, Antel J, Fox RJ, et al. B-cell depletion with rituximab in relapsing-remitting multiple sclerosis. The New England journal of medicine. 2008; 358(7):676–88. doi: 10.1056/NEJMoa0706383 PMID: 18272891.

48. Dearden CE. Alemtuzumab in lymphoproliferate disorders. Reviews in clinical and experimental hematology. 2002; 6(4):435–48; discussion 49–50. PMID: 12823782.

49. Cohen JA, Coles AJ, Arnold DL, Confavreux C, Fox EJ, Hartung HP, et al. Alemtuzumab versus interferon beta 1a as first-line treatment for patients with relapsing-remitting multiple sclerosis: A randomised controlled phase 3 trial. Lancet. 2012; 380(9856):1819–28. doi: 10.1016/S0140-6736(12)61769-3 PMID: 23122652.

50. Morschhauser F, Marlton P, Vitolo U, Linden O, Seymour JF, Crump M, et al. Results of a phase i/ii study of ocrelizumab, a fully humanized anti-cd20 mab, in patients with relapsed/refractory follicular lymphoma. Annals of oncology: official journal of the European Society for Medical Oncology / ESMO. 2010; 21(9):1870–6. doi: 10.1093/annonc/mdq027 PMID: 20157180.

51. Kappos L, Li D, Calabresi PA, O'connor P, Bar-Or A, Barkhof F, et al. Ocrelizumab in relapsing-remitting multiple sclerosis: A phase 2, randomised, placebo-controlled, multicentre trial. Lancet. 2011; 378 (9805):1779–87. doi: 10.1016/S0140-6736(11)61649-8 PMID: 22047971.

52. Lee MK, Cleveland DW. Neuronal intermediate filaments. Annual review of neuroscience. 1996; 19:187–217. doi: 10.1146/annurev.ne.19.030196.001155 PMID: 8833441.

53. Petzold A, Gveric D, Groves M, Schmierer K, Grant D, Chapman M, et al. Phosphorylation and compactness of neurofilaments in multiple sclerosis: Indicators of axonal pathology. Exp Neurol. 2008; 213 (2):326–35. doi: 10.1016/j.expneurol.2008.06.008 PMID: 18619438; PubMed Central PMCID: PMC2583254.

54. Makino S, Kunimoto K, Muraoka Y, Mizushima Y, Katagiri K, Tochino Y. Breeding of a non-obese, diabetic strain of mice. Jikken dobutsu Experimental animals. 1980; 29(1):1–13. PMID: 6995140.

55. Leiter EH. The nod mouse: A model for insulin-dependent diabetes mellitus. Current protocols in immunology / edited by Coligan John E [et al]. 2001;Chapter 15:Unit 15 9. doi: 10.1002/0471142735.im1509s24 PMID: 18432739.

56. Leiter EH, Prochazka M, Coleman DL. The non-obese diabetic (nod) mouse. Am J Pathol. 1987; 128 (2):380–3. PMID: 3303953; PubMed Central PMCID: PMC1899627.

57. Basso AS, Frenkel D, Quintana FJ, Costa-Pinto FA, Petrovic-Stojkovic S, Puckett L, et al. Reversal of axonal loss and disability in a mouse model of progressive multiple sclerosis. The Journal of clinical investigation. 2008; 118(4):1532–43. doi: 10.1172/JCI33464 PMID: 18340379; PubMed Central PMCID: PMC2267014.

58. Mayo L, Trauger SA, Blain M, Nadeau M, Patel B, Alvarez JI, et al. Regulation of astrocyte activation by glycolipids drives chronic cns inflammation. Nature medicine. 2014; 20(10):1147–56. doi: 10.1038/nm.3681 PMID: 25216636; PubMed Central PMCID: PMC4255949.
